# Genome-Wide Gene Expression Analysis Shows AKAP13-Mediated PKD1 Signaling Regulates the Transcriptional Response to Cardiac Hypertrophy

**DOI:** 10.1371/journal.pone.0132474

**Published:** 2015-07-20

**Authors:** Keven R. Johnson, Jessie Nicodemus-Johnson, Mathew J. Spindler, Graeme K. Carnegie

**Affiliations:** 1 Department of Pharmacology, College of Medicine, University of Illinois at Chicago, Chicago, 60612, IL, United States of America; 2 Department of Human Genetics, University of Chicago, Chicago, IL, United States of America; 3 Gladstone Institute of Cardiovascular Disease, 1650 Owens Street, San Francisco, CA, 94158, United States of America; Texas A& M University Health Science Center, UNITED STATES

## Abstract

In the heart, scaffolding proteins such as A-Kinase Anchoring Proteins (AKAPs) play a crucial role in normal cellular function by serving as a signaling hub for multiple protein kinases including protein kinase D1 (PKD1). Under cardiac hypertrophic conditions AKAP13 anchored PKD1 activates the transcription factor MEF2 leading to subsequent fetal gene activation and hypertrophic response. We used an expression microarray to identify the global transcriptional response in the hearts of wild-type mice expressing the native form of AKAP13 compared to a gene-trap mouse model expressing a truncated form of AKAP13 that is unable to bind PKD1 (AKAP13-ΔPKD1). Microarray analysis showed that AKAP13-ΔPKD1 mice broadly failed to exhibit the transcriptional profile normally associated with compensatory cardiac hypertrophy following trans-aortic constriction (TAC). The identified differentially expressed genes in WT and AKAP13-ΔPKD1 hearts are vital for the compensatory hypertrophic response to pressure-overload and include myofilament, apoptotic, and cell growth/differentiation genes in addition to genes not previously identified as affected by AKAP13-anchored PKD1. Our results show that AKAP13-PKD1 signaling is critical for transcriptional regulation of key contractile, cell death, and metabolic pathways during the development of compensatory hypertrophy *in vivo*.

## Introduction

Cardiac hypertrophy is a physiological response to an increased hemodynamic load that initially acts to normalize ventricular wall stress and improve contractile function, but eventually leads to ventricular dysfunction and heart failure. In hypertension, valvular heart disease, ischemic heart disease and idiopathic cardiomyopathy, cardiac hypertrophy increases morbidity and mortality [1–3]. At the molecular level, hypertrophic signals, such as elevated adrenergic activity, evoke transcriptional activation primarily mediated by the transcription factor Myocyte Enhancer Factor-2 (MEF2; [4]). This induces a reprogramming of cardiac gene expression that drives cardiomyocytes toward a growth paradigm referred to as the fetal gene response [5, 6].

The A-kinase anchoring protein (AKAP)-13 has been well documented as a vital component of the MEF2-mediated fetal gene response and the cardiac hypertrophic signaling cascade [7–9]. In addition to other kinases (i.e., PKA and PKC) and signal transduction proteins, AKAP13 binds PKD1 near the C-terminus of the protein [8]. Upon activation by PKC, PKD1 dissociates from AKAP13 and phosphorylates histone deacytelase 5 (HDAC5) in the nucleus, causing HDAC5 dissociation from MEF2 [7, 8, 10]. This activated MEF2 then drives the transcription of fetal genes encoding proteins involved in cellular contraction, energy metabolism, growth, and Ca^2+^ handling [5, 11]. These proteins, while beneficial to the development of compensatory cardiac hypertrophy, ultimately degrade the performance of the heart with eventual progression to heart failure [12].

Ablation of the AKAP13-PKD1 binding domain (AKAP13-∆PKD1) leads to a diminished hypertrophic response in a mouse model for heart failure (i.e. TAC or Angiotensin II) [9]. Altered expression of MEF2 genes were identified in the AKAP13-∆PKD1 mice suggesting that the loss of PKD1 binding to AKAP13 is a key factor in the diminished hypertrophic response observed in this genotype. In support of this finding, a similar phenotype was observed by cardiac-specific deletion of PKD1 [12]. Moreover, this same study shows that these hearts were resistant to hypertrophy induced by trans-aortic constriction (TAC) and Angiotensin II [12]. These studies present strong evidence that AKAP13 anchored PKD1 is a central mediator of cardiac hypertrophy. However the broader transcriptional implications of disrupting this anchored signaling pathway under hypertrophic conditions have yet to be determined.

We hypothesized that AKAP13-∆PKD1 mice will display an altered cardiac transcriptional response to TAC-induced pressure overload due to the deletion of the PKD1 binding domain on AKAP13. In this study, we assess the global transcriptional response in wild type (WT) and AKAP13-∆PKD1 mice following control surgery (sham) and TAC surgery to induce pressure-overload cardiac hypertrophy. While WT and AKAP13-∆PKD1 mice exhibited only minor transcriptional changes following sham surgeries, our results show that AKAP13-PKD1 mice have a significantly and broadly attenuated transcriptional response following hypertrophic induction by TAC. This altered transcriptional response is likely the basis for the lack of compensatory cardiac hypertrophy in the AKAP13-PKD1 mouse model.

## Materials and Methods

All studies were approved by and performed in accordance with the guidelines of the University of Illinois-Chicago animal care and use committee.

### Animals and Experimental Design

Mature male and female mice were generated from MMRRC gene-trap ES cell line CSJ288 (strain genetic background: B6NCr.129P2) on a C57Bl/6 background and has been fully described in [9, 13]. The AKAP13-ΔPKD1 truncation mutant results from specific integration of a β-Geo cassette (β-Galactosidase/neomycin resistance gene) within the endogenous AKAP13 genomic locus, therefore the truncated AKAP13-β-Geo fusion protein is expressed from the endogenous upstream AKAP13 gene promoter.

### Mouse Models of LV Hypertrophy

Transverse aortic constriction (TAC) was carried out to promote pressure overload-induced cardiac hypertrophy in wild type and gene trap mice as previously described [9]. Briefly, age matched mice were anesthetized with sodium pentobarbital (100 mg/kg, ip), placed on a ventilator (Harvard Rodent Ventilator, Harvard Apparatus, Holliston, MA) and core temperature was maintained at 37°C with a heating pad. Stenosis of the transverse aorta was measured using anatomic M-mode echocardiography from mice with satisfactory echocardiographic images of the aortic arch. Percent stenosis was ~ 65% in mice receiving TAC surgery (calculated as the difference between the normal luminal area and the stenotic area, divided by the normal luminal area).

### Transthoracic M-Mode Echocardiography

Echocardiographic measurements were performed for all experimental groups, before and 30 days after sham or TAC surgery. Mice were initially anesthetized with 3% isoflurane and 100% oxygen inhaled in a closed anesthesia chamber. A plane of anesthesia was then regulated by delivery of 1% isoflurane administered through a nose cone with 100% oxygen. Mice were placed in the dorsal decubitus position on a warming pad to maintain normothermia. Transthoracic two-dimensional, M-mode and pulsed Doppler images were acquired with a high-resolution echocardiographic system (VeVo 770, Visual Sonics, Toronto, ON, Canada) equipped with a 30-MHz mechanical transducer. All measurements were taken in compliance with the American Society of Echocardiography guidelines [14]. Results are based on the average of at least three cardiac cycles.

### RNA Isolation and Microarray Analysis

One month after either sham or TAC surgery, mice were euthanized by CO_2_ asphyxiation and cervical dislocation. Ventricular tissue was then removed and mid-papillary sections were dissected from each heart. Tissue samples were dounce-homogenized in Trizol reagent (Invitrogen, Carlsbad, CA), and total RNA was isolated according to the manufacturer’s guidelines. Total RNA was further purified using RNeasy spin columns (Qiagen, Maryland USA) and subjected to DNAse1 digestion (Qiagen kit). RNA yield and integrity were determined using an Agilent 2100 Bioanalyzer (Agilent Tehnologies). Only samples without evidence of RNA degradation (RNA integrity number >8) were used for further experiments. Genome-wide transcriptional profiles were obtained by hybridizing RNA from 32 samples to the Illumina mouse Ref. 8 v2.0 BeadChip at the UCLA Neuroscience Genomics core. Microarray analyses were performed in R, using the package Lumi [15]. Probe level raw intensity values were log transformed and normalized across arrays using quantile normalization [16]. Probe intensities not sufficiently above background levels (detection pvalue < 0.01) and those that mapped to sex chromosomes were excluded from further analysis, this reduced the number of probes from 25,697 to 24,979. Many genes are represented by more than one probe on the Illumina arrays, therefore we selected the median probe intensity for each gene to represent the gene transcript abundance, the median probe intensity for 8,902 genes were used for downstream analyses.

We then used principle component analysis (PCA) to identify and regress out components that explain an appreciable proportion of the variation in gene expression levels. Chip and euthanization date were significant principle components and were removed using CoMBAT [17]. Further analyses were performed on the residuals after CoMBAT adjustment. Additionally, one individual was identified as an outlier in PCA analyses and was removed (data not shown).

We identified genes that were differentially expressed between WT TAC and WT sham mice, as well as between AKAP13-ΔPKD1 TAC and sham mice and WT sham and AKAP13-ΔPKD1 sham mice using a standard t-test. We adjusted for multiple testing using the false discovery rate approach of Benjamini and Hochburg [18]. The raw data set has been deposited with the NCBI Gene Expression Omnibus.

### Quantitative PCR Validation of Microarray Data

Two step RT-qPCR using SYBR Green I was used to confirm differential gene expression of the following 11 transcripts: Aqp8, TLR-4, LmnA, MybpC2, Nuak1, Ogfrl1, Tgfβ3, TgfβR-3, Desmin, Tnnt1, and Tnnt2. Quantitative PCR was performed as previously reported [9]. Briefly, total RNA was extracted from frozen mouse cardiac tissue using Trizol (Molecular Research Center, Cincinnati, OH, USA). Random-primed, reverse transcribed cDNA synthesis reactions were performed using a High Capacity cDNA Reverse Transcription Kit (Applied Biosystems). Forward and reverse primers were generated based on previously published primer pairings available at PrimerBank (http://pga.mgh.harvard.edu/primerbank/index.html) or Real Time PCR Primer Sets (http://www.realtimeprimers.org/). Primers were analyzed for secondary structure formation using IDT SciTools (www.idtdna.com). Target amplicons were selected between 100 and 150 base pairs with at least one primer spanning an exon junction. Final concentrations of sense and antisense primers were determined for each primer pair based on optimal amplification efficiency and melt curve analysis. Primers are listed in S1 Table. Reactions were carried out with Power SYBR-green PCR mix (Life Technologies) using an Applied Biosystems ViiA7 Real Time PCR system. The Ct (defined as the cycle number at which the fluorescence exceeds the threshold level) was determined for each reaction and quantification was determined using the ∆∆Ct method [19]. The target Ct was determined for each sample and then normalized to the GAPDH mRNA Ct from the same sample. These values were then compared with control levels using the 2^−∆∆Ct^ method and represented as fold difference normalized to the appropriate control sample (*i*.*e*., wild-type sham surgery).

## Results

### AKAP13-∆PKD1 Mice Display an Attenuated Phenotypic and Transcriptional Response to Pressure-Overload Cardiac Hypertrophy

Induction of cardiac hypertrophy in WT mice resulted in a significant increase in heart weight to body weight (HW/BW) ratio as well as left ventricular posterior wall (LVPW) thickness (S1 Fig). There were no significant differences in HW/BW or LVPW thickness in AKAP13-∆PKD1 mice, as these animals failed to develop compensatory cardiac hypertrophy. There are no significant differences in heart rate and blood pressure between WT and AKAP13-ΔPKD1 mice, as established for this model previously [9]. Established transcriptional responses to cardiac hypertrophy were also examined to validate the hypertrophic model for this study. Significantly elevated expression levels of Atrial Natriuretic Peptide (ANP) and Toll-like receptor 4 (TLR-4) were seen in WT mice but not in AKAP13-∆PKD1 mice (S1 Fig). Protein levels of the hypertrophic markers α-actinin and myosin heavy chain-β (Myh-7) were also measured (S2 Fig). Western analysis shows elevated levels of α-actinin and Myh-7 in WT-TAC hearts, but not in AKAP13-∆PKD1 TAC hearts.

#### WT and AKAP13-∆PKD1 mice have similar transcriptional profiles following sham surgeries

**Fig 1:**
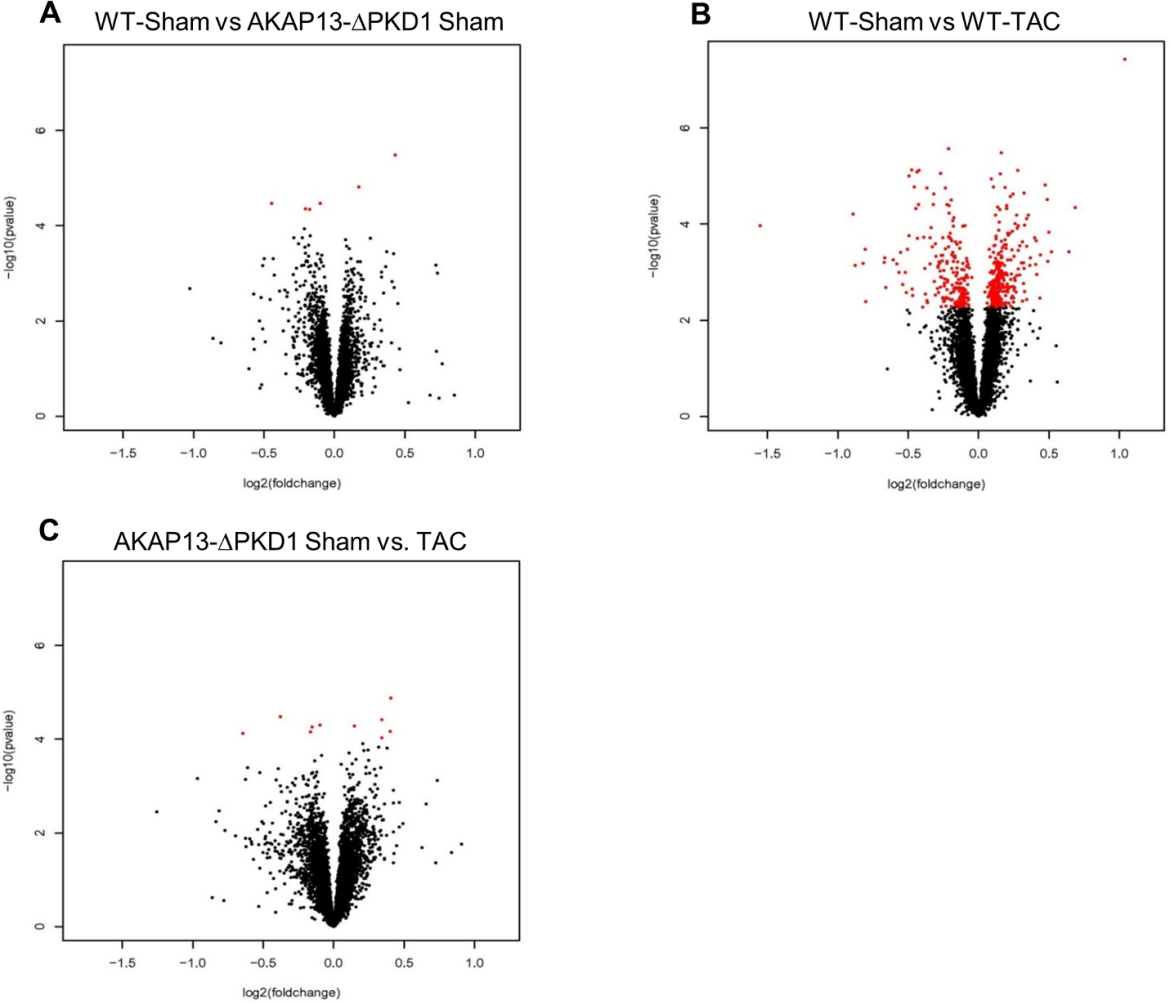
Volcano plot comparative expression analysis of WT and AKAP13-∆PKD1 Sham and TAC mice. The x-axis is the fold-change and the Y-axis is p-value in–log10 scale computed using program R. **A)** The expression of 6 genes were significantly changed in AKAP13-∆PKD1 sham mice compared to wild type sham mice, 4 genes were down-regulated (Crsp 3, Prkab1, Rccd1, and Zfp592) and 2 were up-regulated (Sertad 3 and Lrtm1). Red dots indicate significant changes in gene expression while black dots are not significant. **B)** The expression of 479 probes was significantly changed (>2.5-fold) in WT-TAC mice compared to WT-Sham mice (p<0.05). A total of 204 probes were up-regulated and 275 were down-regulated. **C)** The expression of 10 probes were significantly changed (>2.5-fold) in AKAP13-∆PKD1-TAC mice compared to AKAP13-∆PKD1 sham mice (p<0.05). A total of 5 probes were up-regulated and 5 were down-regulated.

To identify transcriptional changes resultant from the PKD1 anchoring domain deletion on AKAP13, transcriptional profiles of WT-sham and AKAP13-∆PKD1 sham hearts were compared (Fig 1A). Six genes were significantly differentially expressed (FDR <10%) between WT-sham and AKAP13-∆PKD1-sham mice. Two transcripts (Sertad3 and Lrtm 1) had elevated expression in WT-sham compared to AKAP13-∆PKD1 and four transcripts (Crsp3, Prkab1, Rccd1, and Zfp592) had decreased expression in WT-sham compared to AKAP13-∆PKD1 sham (Fig 1A). These genes functions and differential expressions are listed in Table 1.

**Table 1:** Differentially expressed genes between WT-Sham and AKAP13-ΔPKD1 Sham hearts.

Gene Name	Function	Fold Change	p-value
Crsp3	Transcription Factor	-11.15	3.38X10^-5^
Prkab1	AMPK regulatory subunit	-5.14	4.43X10^-5^
Rccd1	unknown	-2.52	3.37X10^-5^
Sertad3	Transcriptional co-activator	4.34	1.52X10^-5^
Zfp592	Transcriptional regulation	-4.37	4.51X10^-5^
Lrtm 1	Integral Membrane protein	5.62	3.3X10^-6^

### Abnormal Transcriptional Response to TAC in Hearts of AKAP13-∆PKD1 Mice

In WT hearts TAC induction was associated with up-regulation of 204 transcripts and down-regulation of 275 transcripts that were not differentially expressed in AKAP13-∆PKD1 TAC mice (Fig 1B, S2 Table). Similarly regulated transcripts between previous studies utilizing TAC-induced cardiac hypertrophy and this study include Acta1, MybpC2/3, Actn2, and Myh7 among others indicating an appropriate hypertrophic gene transcriptional response in WT mice.

To assess the extent to which AKAP13-mediated PKD1 anchoring deletion influences hypertrophic transcriptional response, AKAP13-∆PKD1 TAC hearts were compared to AKAP13-∆PKD1 sham hearts. In AKAP13-∆PKD1 hearts, after one month of TAC significant transcriptional changes were identified for 10 genes, 5 were up-regulated and 5 down-regulated (Fig 1C). Seven of these 10 genes were uniquely differentially expressed in AKAP13-∆PKD1 TAC mice (Scara5, Alox12, Sh3g12, Cish, Gtf2e2, Snapap, and Prmt7) and are compared in Table 2. Of these 10 genes, three transcripts were similarly regulated in WT-TAC and AKAP13-∆PKD1 TAC (Nrap, Ankrd1, and Admr).

**Table 2:** Differentially expressed genes between AKAP13-ΔPKD1-Sham and AKAP13-ΔPKD1 TAC hearts.

Gene Name	Function	Fold Change	p-value
Scara5	Mediates cellular uptake of ferritin-bound iron	-10.2	1.32X10^-5^
Gtf2e2	Influences RNA polymerase II activity.	4.1	7.04X10^-5^
Snapap	Component of BLOC-1 complex	3.8	5.55X10^-5^
Alox12	Oxygenase and leukotriene A4 synthase activity	-8.55	3.82X10^-5^
Sh3gl2	Involved in synaptic vesicle endocytosis.	-10.07	6.87X10^-5^
Cish	regulation of cytokines signaling	-8.6	9.4X10^-5^

### Multiple Signaling Networks Are Activated in WT TAC-Induced Hypertrophy but Are Absent in AKAP13-∆PKD1 Hearts

**Fig 2:**
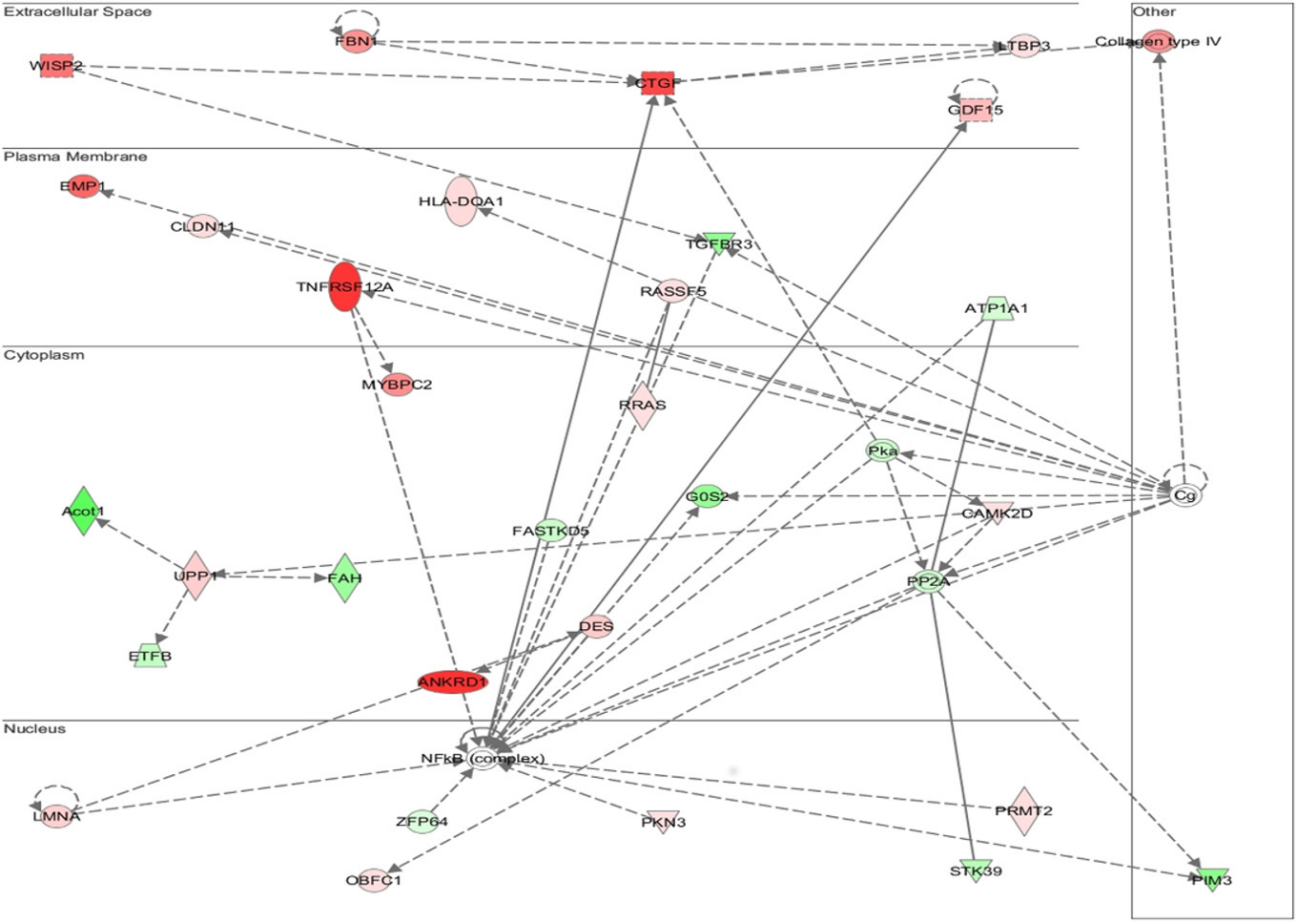
IPA Network analysis of differentially regulated genes. A top modified gene network modified in WT-TAC mice that is not modified in AKAP13-∆PKD1 mice is cell death and survival, cell movement, and organismal development. Genes shaded in red are up-regulated while genes shaded in green are down-regulated.

**Fig 3:**
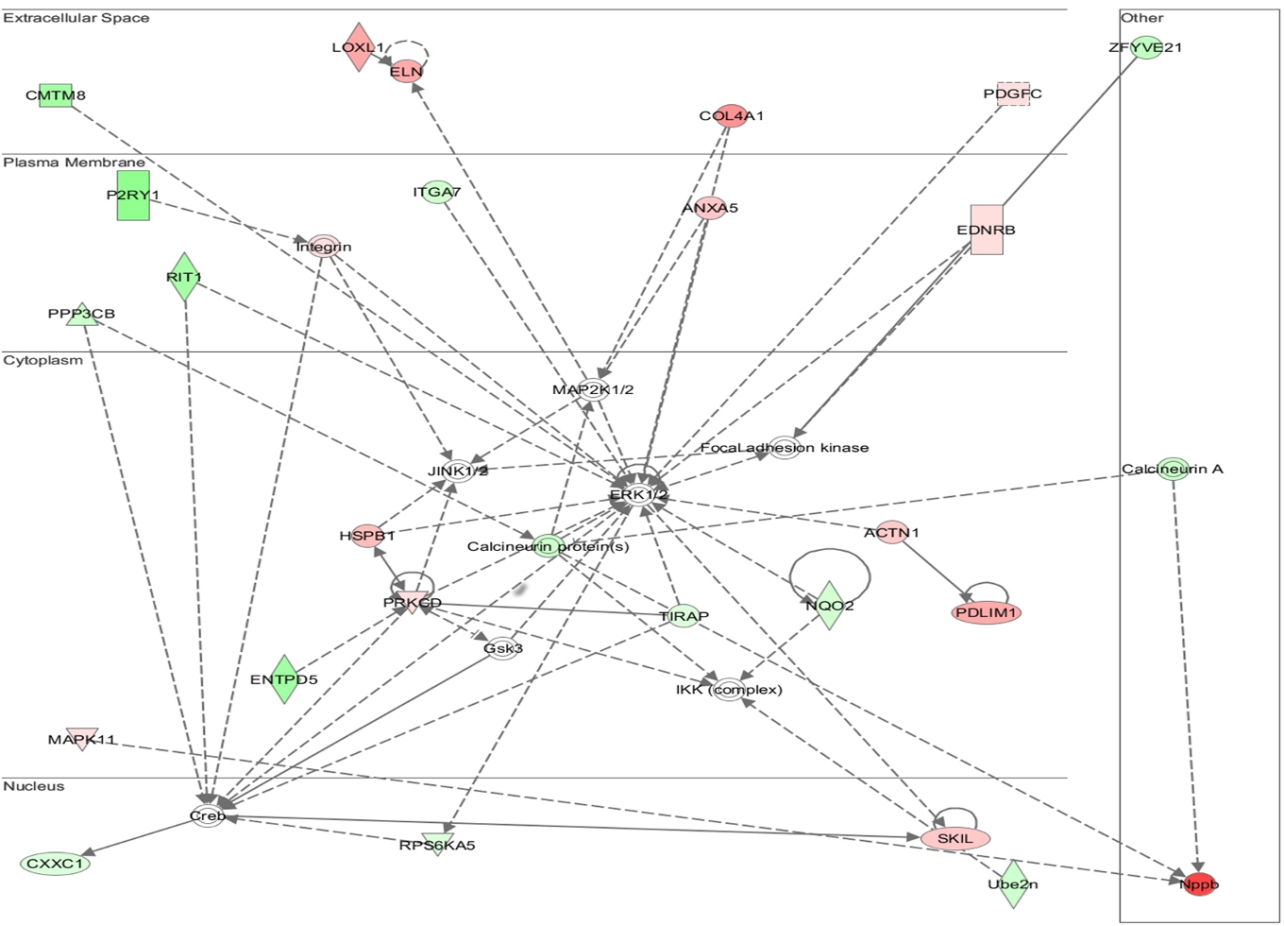
IPA Network analysis of differentially regulated genes. A top modified gene network modified in WT-TAC mice that is not modified in AKAP13-∆PKD1 mice is cardiovascular system development, tissue morphology and cell movement. Genes shaded in red are up-regulated while genes shaded in green are down-regulated.

**Fig 4:**
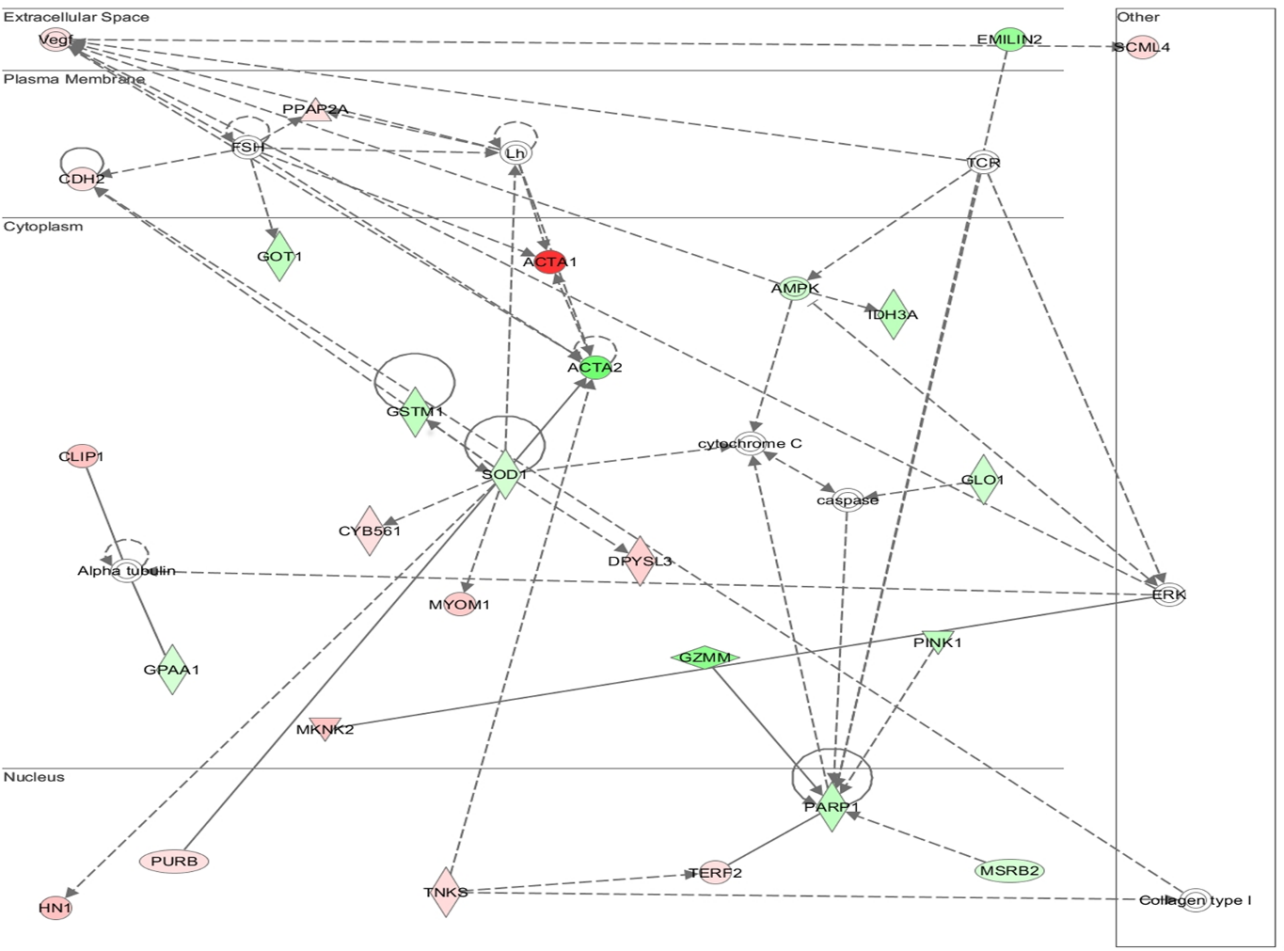
IPA Network analysis of differentially regulated genes. A top modified gene network modified in WT-TAC mice that is not modified in AKAP13-∆PKD1 mice is drug metabolism, protein synthesis, and neurological disease. Genes shaded in red are up-regulated while genes shaded in green are down-regulated.

**Fig 5:**
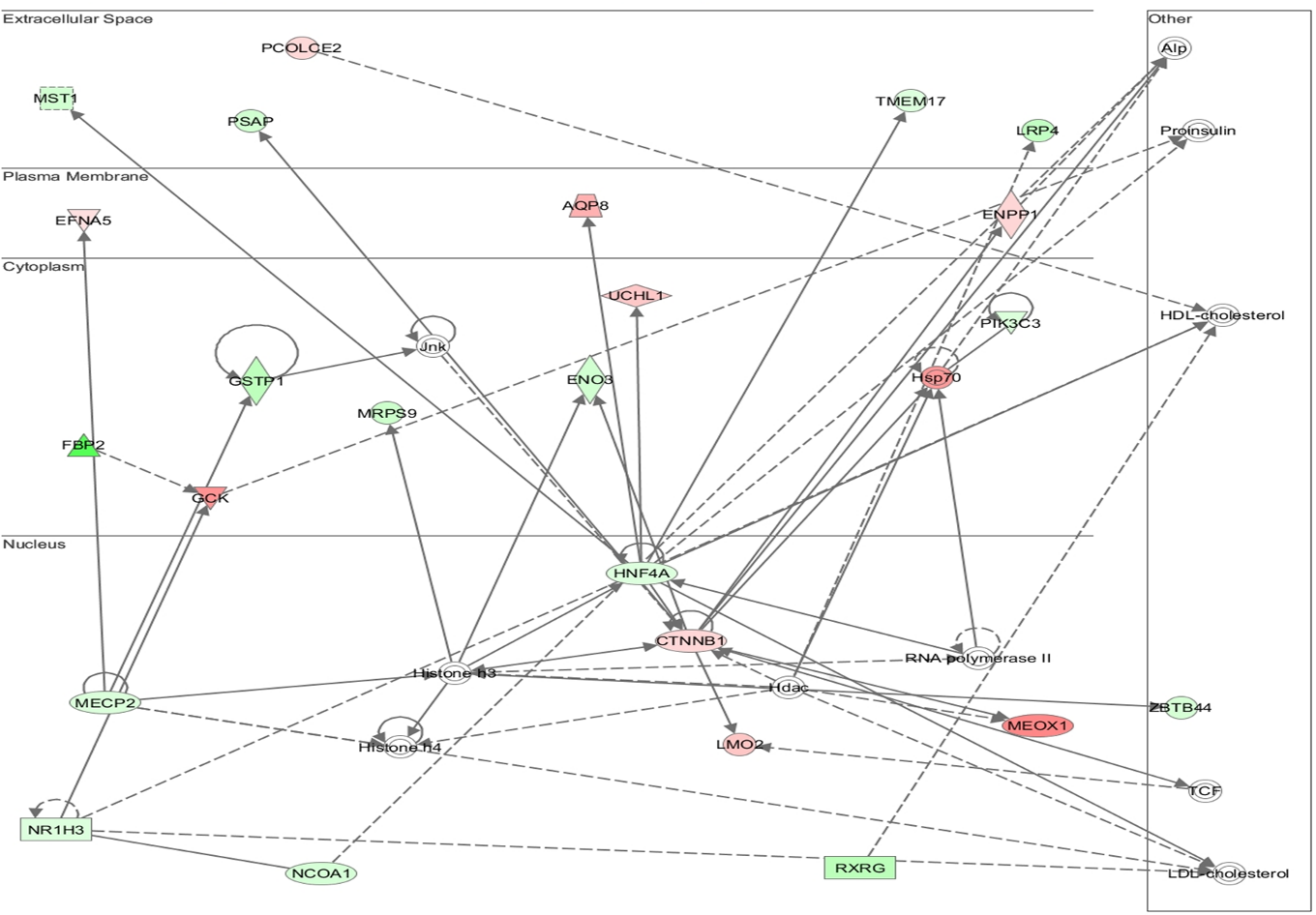
IPA Network analysis of differentially regulated genes. A top modified gene network modified in WT-TAC mice that is not modified in AKAP13-∆PKD1 mice is lipid metabolism, molecular transport, and small molecule biochemistry. Genes shaded in red are up-regulated while genes shaded in green are down-regulated.

**Fig 6:**
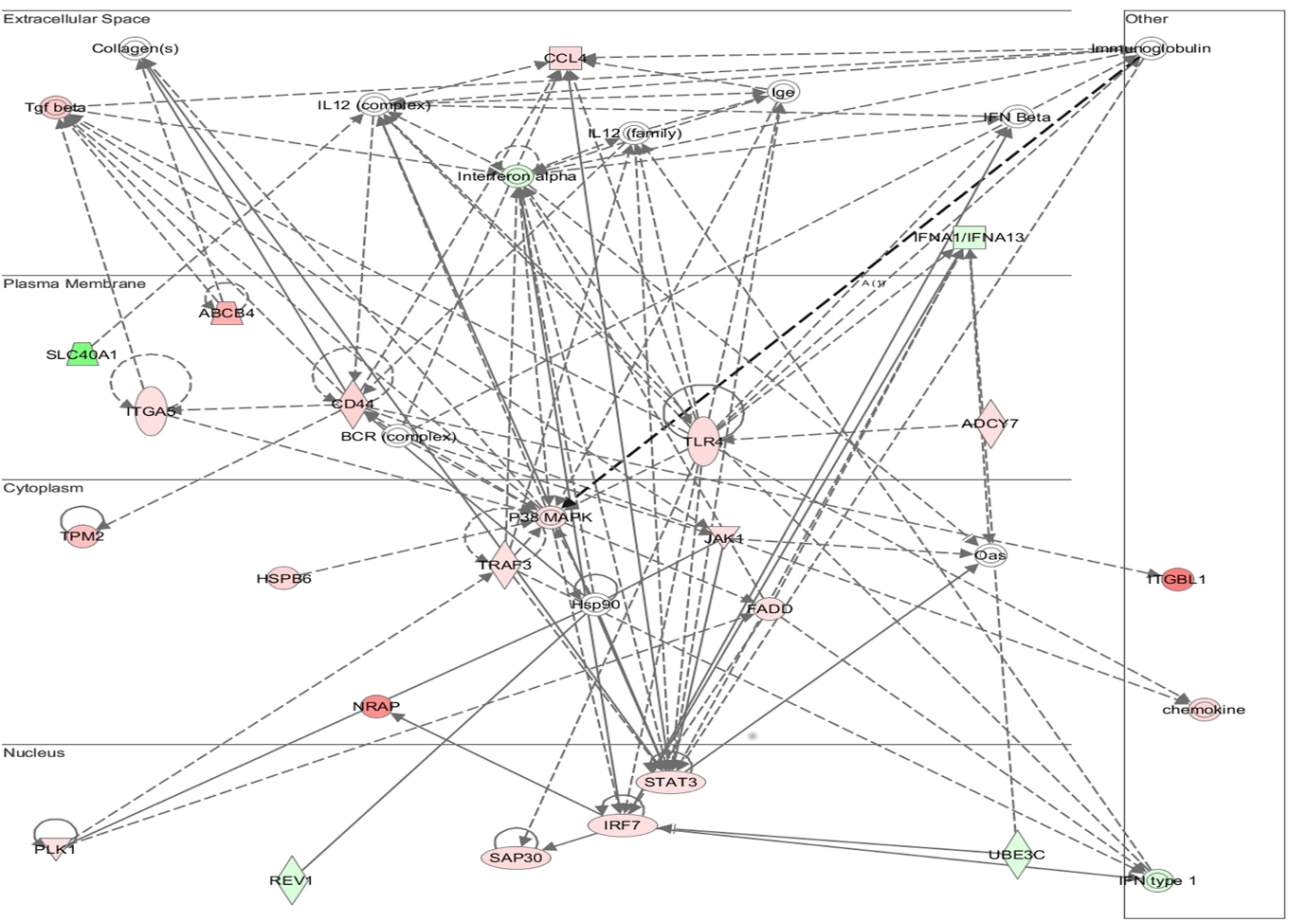
IPA network analysis of differentially regulated genes. A top modified gene network modified in WT-TAC mice that is not modified in AKAP13-∆PKD1 mice is neurological disease, psychological disorders, skeletal and muscular disorders. Genes shaded in red are up-regulated while genes shaded in green are down-regulated.

**Fig 7:**
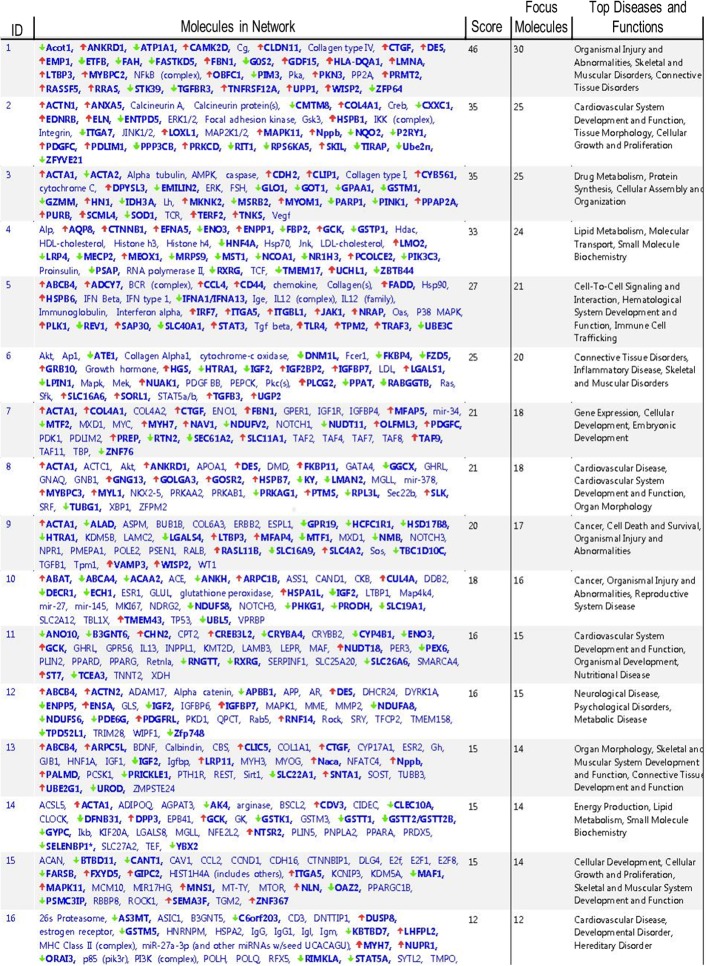
Functional gene networks and associated functions and diseases modified following TAC-induction in WT hearts but not in AKAP13-ΔPKD1 TAC hearts.

Functional annotation and interrogation via the Ingenuity Pathway Analysis (IPA) suite identified multiple functional gene groupings differentially regulated in WT-TAC that were not altered in AKAP13-∆PKD1 mice (Figs 2–6, Table 3). The top molecular and cellular canonical functions identified included (in descending order of significance): cell death and survival, drug metabolism, protein synthesis, cellular development, and cellular growth and proliferation. The top representative physiological systems development and functions are organ morphology, skeletal and muscular system development and function, cardiovascular system development and function, tissue morphology, and hepatic system development and function. The top represented diseases and disorders include neurological disease, cancer, gastrointestinal disease, psychological disorders, and skeletal and muscular disorders. Network analysis identified significantly and uniquely modified networks in WT-TAC hearts that were not modified in AKAP13-∆PKD1 TAC hearts (Table 3). Additionally, functional gene networks (and constituent genes) modified following TAC-induction in WT mice but not in AKAP13-∆PKD1 mice are shown in Fig 7. Top upstream regulators (S4 Table), and toxicity functions (S5 Table) were also identified in network analyses.

**Table 3:** Top functional gene groupings activated in WT-TAC but not AKAP13-ΔPKD1 TAC hearts.

Molecular and Cellular Functions	p-value Range	No. Genes
Cell Death and Survival	2.73x10^-7^–1.69x10^-2^	92
Drug Metabolism	7.91x10^-7^–1.68x10^-2^	112
Protein Synthesis	7.91x10^-7^–1.67x10^-2^	66
Cellular Development	2.65x10^-6^–1.69x10^-2^	78
Cellular Growth and Proliferation	9.41x10^-6^–1.59x10^-2^	98
**Physiological Systems Development and Function**		
Organ Morphology	9.41x10^-6^–1.59x10^-2^	63
Skeletal and Muscular System Development and Function	5.05x10^-6^–1.67x10^-2^	52
Cardiovascular System Development and Function	1.20x10^-5^–1.63x10^-2^	63
Tissue Morphology	1.94x10^-5^–1.53x10^-2^	36
Hepatic System Development and Function	1.94x10^-5^–1.53x10^-2^	26
**Diseases and Disorders**		
Neurological Disease	2.73x10^-7^–1.69x10^-2^	92
Cancer	7.91x10^-6^–1.68x10^-2^	112
Gastrointestinal Disease	7.91x10^-7^–1.67x10^-2^	66
Psychological Disorders	2.65x10^-6^–1.59x10^-2^	78
Skeletal and Muscular Disorders	9.41x10^-6^–1.59x10^-2^	98

### Expression Levels of Key Genes Involved in Contraction, Apoptosis, Cell Growth, Energy Metabolism, and Oxidative Stress Are Attenuated in AKAP13-∆PKD1 Response to TAC

**Fig 8:**
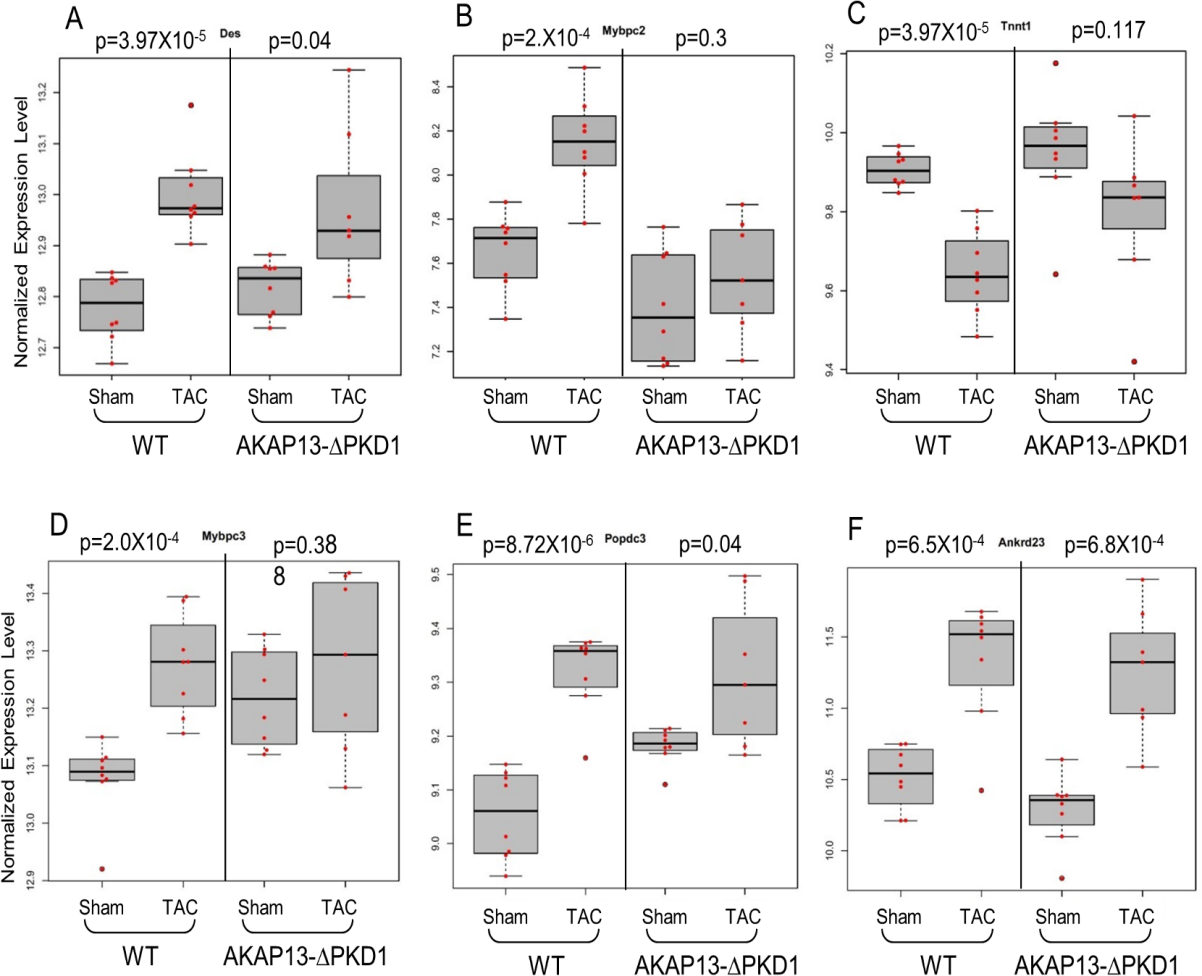
Myofilament Gene expression stratified by WT or AKAP13-∆PKD1 mice following sham or TAC surgery. Normalized (log-transformed) gene expression is shown for myofilament proteins **A)** Desmin (Des), **B)** Myosin binding protein C-2 (MybpC2), **C)** Troponin T1 (Tnnt1), **D)** Myosin binding protein C-3, **E)** Popeye protein-3 (Popcd3), and **F)** Ankyrin repeat domain 23 (Ankrd23). Gray boxes represent the interquartile range, encompassing the first through third quartiles; the horizontal bar shows the median value. Values greater than 1.5 times the interquartile range are plotted outside of the whiskers. P values are from linear regression assuming an additive model.

**Fig 9:**
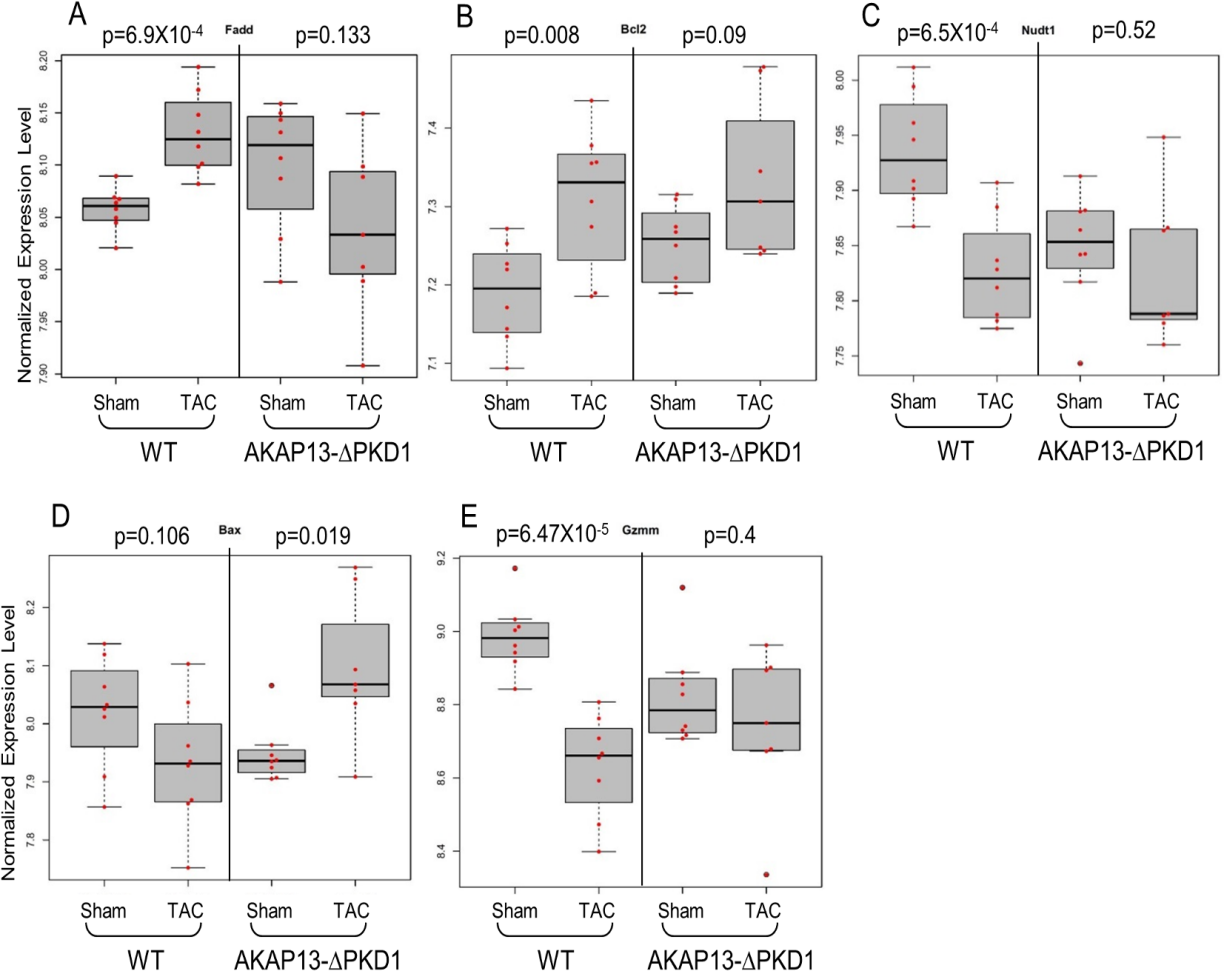
Apoptosis Gene expression stratified by WT or AKAP13-∆PKD1 mice following sham or TAC surgery. Normalized gene expression is shown for apoptosis-associated genes **A)** Fas-associated death domain (FADD), **B)** Bcl2, **C)** Nudt1 **D)** Bax, **E)** Granzyme M (Gzmm), and **F)** Dynamin-1 like protein. Gray boxes represent the interquartile range, encompassing the first through third quartiles; the horizontal bar shows the median value. Values greater than 1.5 times the interquartile range are plotted outside of the whiskers. P values are from linear regression assuming an additive model.

**Fig 10:**
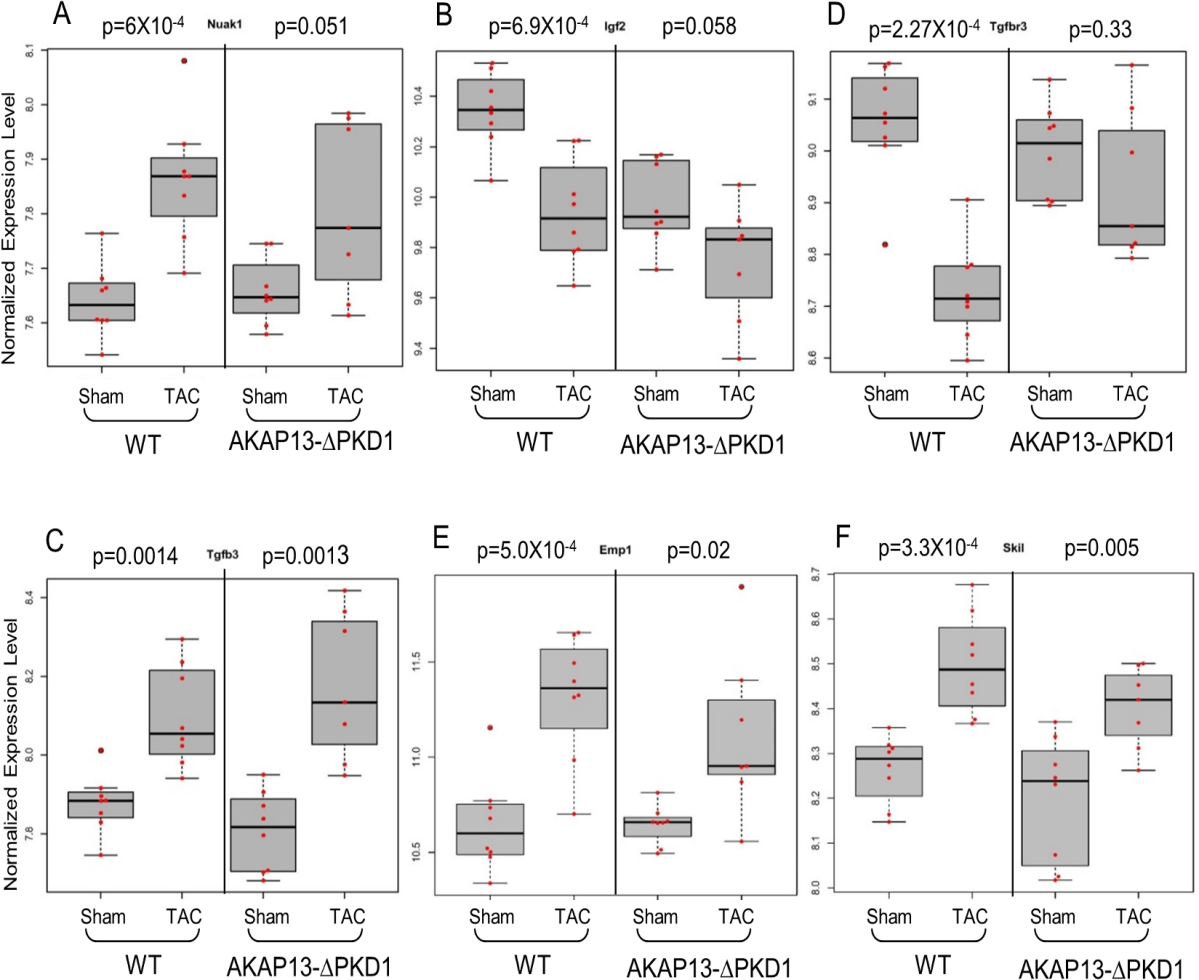
Cell Growth/Differentiation Gene expression stratified by WT or AKAP13-∆PKD1 mice following sham or TAC surgery. Normalized gene expression is shown for cellular growth and differentiation genes **A)** Nuak1, **B)** Igf2, **C)** TgfβR-3 **D)**, Tgfβ-3 **E)**, Emp1 and **F)** Skil. Gray boxes represent the interquartile range, encompassing the first through third quartiles; the horizontal bar shows the median value. Values greater than 1.5 times the interquartile range are plotted outside of the whiskers. P values are from linear regression assuming an additive model.

**Fig 11:**
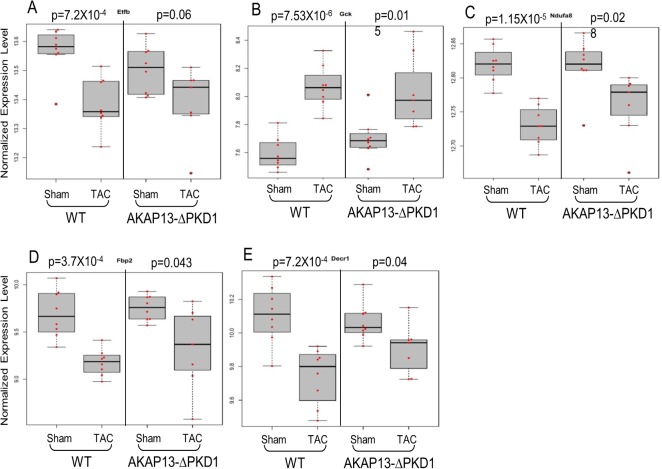
Metabolism gene expression stratified by WT or AKAP13-∆PKD1 mice following sham or TAC surgery. Normalized gene expression is shown for cellular metabolism-associated genes **A)** Etfb, **B)** Gck, **C)** Ndufa8, **D)** Fbp2, **E)** Decr1. Gray boxes represent the interquartile range, encompassing the first through third quartiles; the horizontal bar shows the median value. Values greater than 1.5 times the interquartile range are plotted outside of the whiskers. P values are from linear regression assuming an additive model.

**Fig 12:**
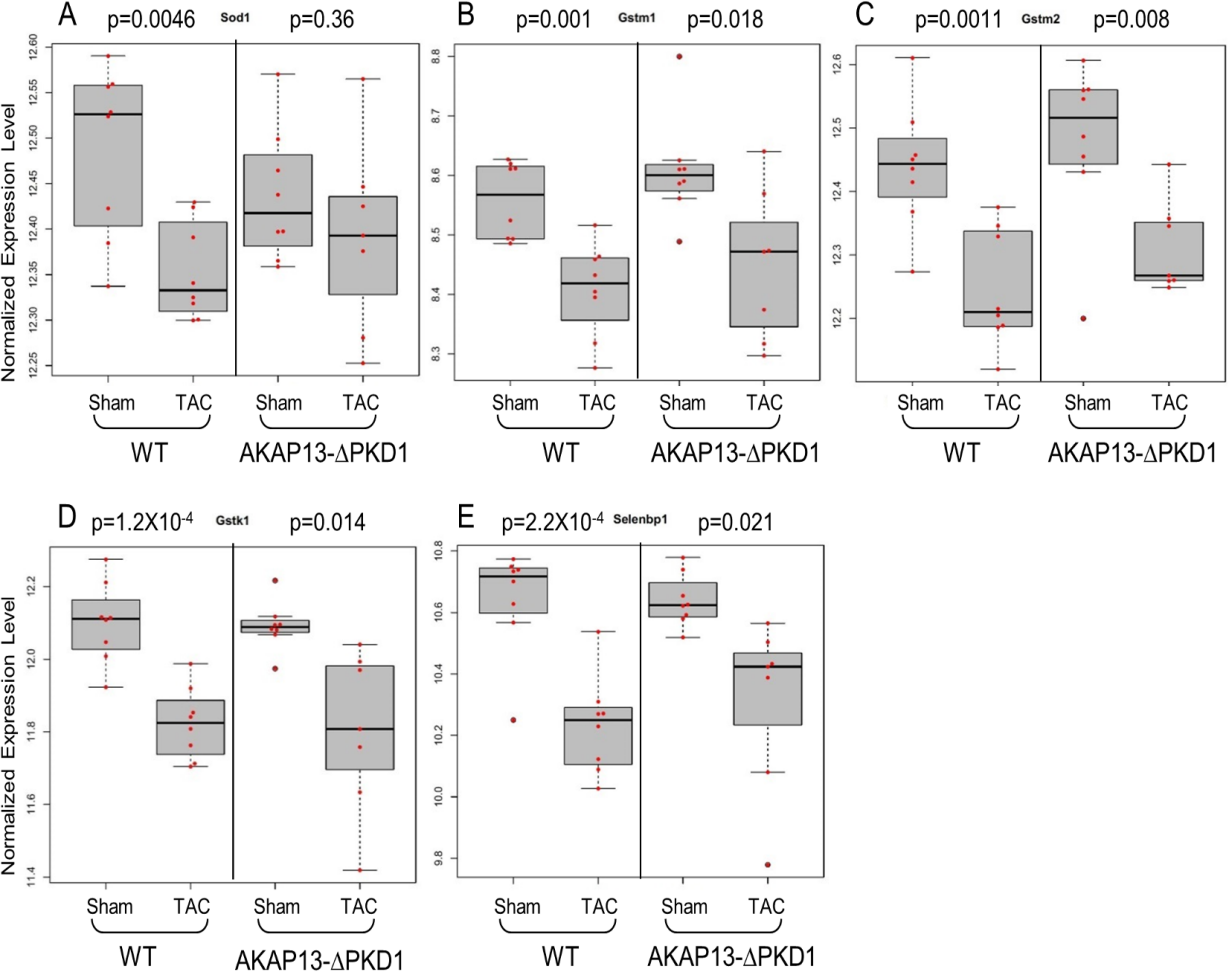
Oxidative stress gene expression stratified by WT or AKAP13-∆PKD1 mice following sham or TAC surgery. Normalized gene expression is shown for oxidative stress response-associated genes **A)** Sod1, **B)** Gstm1, **C)** Gstm2, **D)** Gstk1, and **E)** Selenbp1. Gray boxes represent the interquartile range, encompassing the first through third quartiles; the horizontal bar shows the median value. Values greater than 1.5 times the interquartile range are plotted outside of the whiskers. P values are from linear regression assuming an additive model.

Select gene differential expression changes identified via microarray interrogation of WT and AKAP13-∆PKD1 hearts were sub-categorized into myofilament, apoptosis, cell growth/differentiation, energy metabolism, or oxidative stress–related molecules (Figs 8–12). Myofilament-Associated Genes: Significant differences between WT-sham/TAC and AKAP13-∆PKD1-Sham/TAC were seen in multiple myofilament genes (Fig 8). Desmin (Des), myosin binding protein C-2 (MybpC-2), troponin T-1 (Tnnt1), and myosin binding protein C-3 (MybpC3) were all significantly different between WT-Sham and WT-TAC hearts but not between AKAP13-∆PKD1-sham and TAC hearts. The cardiac muscle development-associated gene Popdc3 is elevated in both WT and AKAP13-∆PKD1 TAC hearts compared to sham, though at different significance level (Fig 8E). The stretch-induced transcription factor Ankrd23 is elevated in both WT-TAC and AKAP13-∆PKD1 TAC compared to sham hearts (Fig 8F). Apoptosis-Associated Genes: Significantly elevated apoptosis gene expression levels were identified in WT-TAC but not GT-TAC for Fadd and Bcl2 (Fig 9A and 9B). Expression levels for the genes Bax, Nudt1, and Gzmm are significantly reduced in WT-TAC but not AKAP13-∆PKD1 TAC compared to sham (Fig 9C–9E). Cell growth and differentiation-associated genes: Significantly elevated expression of Nuak1 was seen in WT-TAC but not GT-TAC (Fig 10A), while significantly reduced expression of Igf2 and Tgfβr3 was seen in WT-TAC but not AKAP13-∆PKD1 TAC (Fig 10B and 10C). Significantly elevated expression of both Tgfb3 and Emp1 are seen in WT-TAC and AKAP13-∆PKD1 TAC (Fig 10D and 10E). Metabolism-associated genes: Significant reduction in Etfb expression was found in WT-TAC but not AKAP13-∆PKD1 TAC hearts (Fig 11A). Several other energy metabolism-related genes were significantly altered in both WT-TAC and AKAP13-∆PKD1 TAC hearts, though at different significance levels (Fig 11B–11E). Oxidative stress response genes: Expression of the oxidative stress-response gene Sod1 was significantly reduced in WT-TAC hearts but not in AKAP13-∆PKD1 TAC hearts (Fig 12A). Other oxidative stress-response genes had similar expression profiles for WT-TAC and AKAP13-∆PKD1 TAC hearts (Fig 12B–12E).

A subset of the above gene transcriptional changes was further validated by qPCR analysis. As shown in S3 Fig, genes assessed by qPCR exhibited expression changes during TAC induction that were consistent with the responses detected by microarray analysis.

## Discussion

Various types of signals stimulate the induction of cardiac hypertrophy, which is accompanied by transcriptional reprogramming of cardiac gene expression. In this study we have identified a large number of genes and associated pathways that are differentially expressed in the normal hypertrophic response (WT-TAC mice) but are largely attenuated in AKAP13-PKD1 TAC mice. These genes are vital to several aspects of the compensatory cardiac hypertrophic response and are involved in such processes as cardiac myofilament contractility, apoptosis, energy metabolism, cell growth/differentiation, and oxidative stress.

As shown previously [7, 20], the C-terminus of AKAP13 provides an anchoring domain for PKD1, which upon activation by PKC, translocates to the nucleus and phosphorylates HDAC5 and activates the MEF2-mediated fetal gene response [12, 21]. In addition to MEF2 de-repression and fetal gene activation, activated PKD1 has also been shown to mediate cardiac transcription through phosphorylation of cAMP Response Element Binding protein (CREB) [22]. In the present study, activation of the CREB-target gene Bcl-2 was seen in WT-TAC hearts but not AKAP13ΔPKD1-TAC hearts (Fig 9) suggesting PKD1 transcriptional regulation independent of MEF2 is also blunted in AKAP13ΔPKD1 hearts.

Consistent with a previous study, the AKAP13-∆PKD1 mice used here broadly failed to develop the phenotypic characteristics of compensatory hypertrophy [9]. The role of AKAP13 anchored PDK1 in activating the hypertrophic gene response through MEF2 activation in cardiac myocytes has been well established [7, 9], however the broader transcriptional implications of disrupting AKAP13-PKD1 anchoring have remained unknown until now. Consistent with a previous phenotypic study [13], under basal non-stressed conditions, deletion of the AKAP13 PKD1 anchoring domain had minimal effect on global cardiac gene transcription as only six genes were identified as differentially expressed between WT and gene trap-sham treated animals. Though of these six transcripts, three encode transcription factors (Crsp3/Med23) or co-activators (Sertad3, and Zfp592) and were significantly down-regulated in AKAP13-∆PKD1 hearts. The protein encoded by Crsp3/Med23 gene is involved in the regulated transcription of nearly all RNA polymerase II-dependent genes [23–25], and Sertad3 functions as an activator of cell growth [26]. These genes have not been previously identified as down- stream targets of PKD1 though evidence presented here suggests that AKAP13-mediated PKD1 anchoring is necessary for the basal expression of these genes and may have a role in homeostatic transcriptional regulation.

When challenged with hypertrophic stimuli (i.e., humoral or pressure overload), the contractile features of the heart change in an adaptation to the stress, commonly known as fetal gene reprogramming. In the WT-TAC hearts used in this study, multiple MEF2-associated myofilament protein encoding genes were significantly differentially expressed (i.e., myosin heavy chain α/β, α-actinin, mybpc2/3, des, and myom) as previously reported [27–29]. In contrast AKAP13-∆PKD1 hearts did not exhibit differential expression of these myofilament genes, though the myofilament stretch-responsive transcription factor Ankrd23 [30] was identically expressed in WT and AKAP13-∆PKD1 hearts suggesting that the stretch-sensing mechanism has remained intact.

It is also critical for cardiac muscle to adapt to hypertrophic stress by preventing cell death (apoptosis) and by enhancing cell survival. As seen in this study, WT and AKAP13-∆PKD1 hearts have strikingly different apoptotic-gene expression responses. Pro-apoptotic genes Bax, Gzmm, and Dnm1l have significantly reduced expression in WT-TAC hearts while AKAP13-∆PKD1 hearts have significantly elevated expression of Bax, and unchanged levels of Gzmm and Dnm1l. Expression of the anti-apoptotic gene Bcl2 is significantly increased in WT hearts but not in AKAP13-∆PKD1 hearts. Not surprisingly, one of the top functional gene groupings altered between WT and AKAP13-∆PKD1 hearts is cellular death and survival. Taken together, these findings suggest that AKAP13-∆PKD1 hearts are more susceptible to apoptosis than WT, which is consistent with a previous finding by us that apoptotic levels in AKAP13-∆PKD1 hearts are significantly elevated over WT-TAC hearts [9].

Cardiac metabolism also adapts to the stress of pressure-overload hypertrophy by shifting from fatty acid oxidation to oxidation of glycogen, lactate, and glucose [31–34]. The shifting transcriptional response from genes involved in β-oxidation to those involved in glucose metabolism is readily seen in the WT hearts but not AKAP13-∆PKD1 hearts used in this study. Reduced expression of the oxidative metabolism genes Etfb, Ndufa8, and Decr1, and elevated expression of hexokinase (Gck) was seen in WT hearts but not AKAP13-∆PKD1 hearts and indicates that PKD1 anchoring through AKAP13 is at least partially necessary for this metabolic switch during the development of compensatory hypertrophy.

## Supporting Information

S1 Fig Echocardiographic and gravimetric characteristics of hearts from AKAP13-∆PKD1 mice and control WT littermates. Echocardiographic measurements were taken at baseline and 1 month following sham or TAC surgery. **A)** One month after TAC surgery, WT mice have a significant increase in heart weight (HW) to body weight (BW) ratio (p<0.01). AKAP13-∆PKD1 mice have similar HW/BW ratios compared between sham and TAC surgeries. **B)** Left ventricular posterior wall thickness (LVPW) in diastole is significantly increased in WT-TAC mice compared to baseline measurements (*p<0.05). **C)** Cardiac ANP expression is significantly elevated in WT-TAC mice compared to sham. Left panel; expression levels determined by microarray, Right panel; expression levels determined by quantitative PCR. No difference was seen between AKAP13-∆PKD1-sham and TAC animals. **D)** Cardiac Toll-like receptor-4 (TLR4) expression was significantly elevated in WT-TAC animals compared to sham. Left panel; expression levels determined by microarray, Right panel; expression levels determined by quantitative PCR. TLR4 expression was also significantly elevated in AKAP13-∆PKD1 sham animals compared to WT-sham.
(PPT)

S2 Fig Western analysis of hypertrophic marker proteins. Protein levels of **A)** α-actinin and **B)** myosin heavy chain-β were significantly elevated in WT-TAC hearts, indicative of a normal hypertrophic response. These proteins were not significantly different in AKAP13-∆PKD1 sham/TAC hearts.
(PPTX)

S3 Fig Validation of microarray expression data by qPCR. Shown are expression changes determined via qPCR analysis for **A)** Aqp8, **B)** LMNA, **C)** MybpC2, **D)** NUAK1, **E)** Tgfβ-3, **F)** TnnT1, **G)** TnnT2, **H)** Ogfrl1.
(PPTX)

S1 Table Primers used in RT-PCR validation analyses. (DOC)

S2 Table Significant differentially expressed genes identified in WT-TAC hearts that are not significant in AKAP13-ΔPKD1 TAC hearts. (XLS)

S3 Table Top canonical pathways. (DOC)

S4 Table Top upstream regulators. (DOC)

S5 Table Top toxicity functions-cardiotoxicity. (DOC)

S6 Table Significant differentially expressed genes between WT-TAC and AKAP13-ΔPKD1 TAC hearts. (XLSX)
