# Supplementary material for: Genome-Wide Gene Expression Analysis Shows AKAP13-Mediated PKD1 Signaling Regulates the Transcriptional Response to Cardiac Hypertrophy
Source: PLoS One. 2015 Jul 20;10(7):e0132474. doi: 10.1371/journal.pone.0132474 (PMC4508115; doi:10.1371/journal.pone.0132474)
Supplement: S1 Fig — Echocardiographic measurements were taken at baseline and 1 month following sham or TAC surgery. A) One month after TAC surgery, WT mice have a significant increase in heart weight (HW) to body weight (BW) ratio (p<0.01). AKAP13-∆PKD1 mice have similar HW/BW ratios compared between sham and TAC surgeries. B) Left ventricular posterior wall thickness (LVPW) in diastole is significantly increased in WT-TAC mice compared to baseline measurements (*p<0.05). C) Cardiac ANP expression is significantly elevated in WT-TAC mice compared to sham. Left panel; expression levels determined by microarray, Right panel; expression levels determined by quantitative PCR. No difference was seen between AKAP13-∆PKD1-sham and TAC animals. D) Cardiac Toll-like receptor-4 (TLR4) expression was significantly elevated in WT-TAC animals compared to sham. Left panel; expression levels determined by microarray, Right panel; expression levels determined by quantitative PCR. TLR4 expression was also significantly elevated in AKAP13-∆PKD1 sham animals compared to WT-sham. (PPT) [file pone.0132474.s001.ppt]

## Slide 1
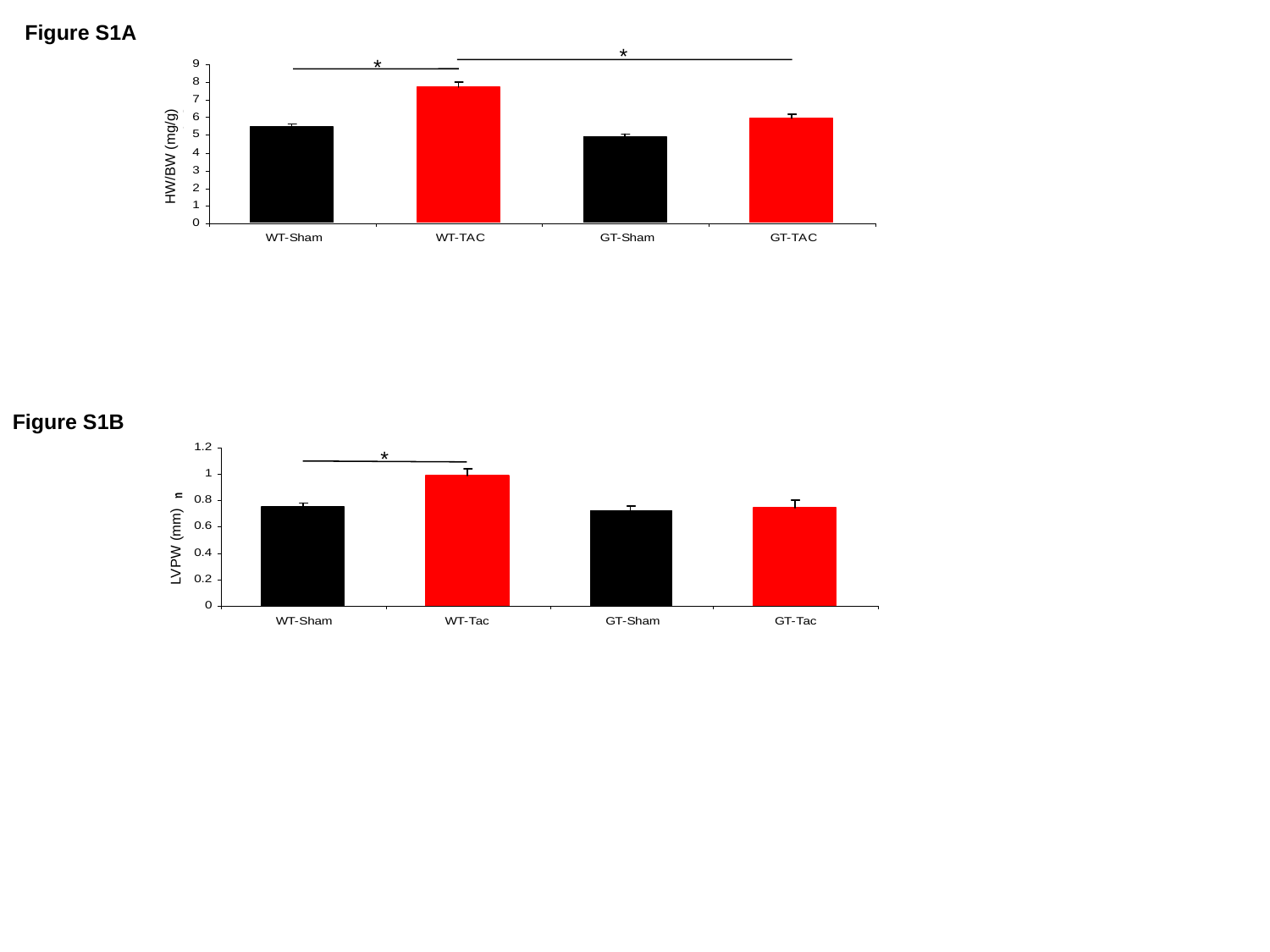

Figure S1A
*
*
HW/BW (mg/g)
Figure S1B
*
LVPW (mm)

## Slide 2
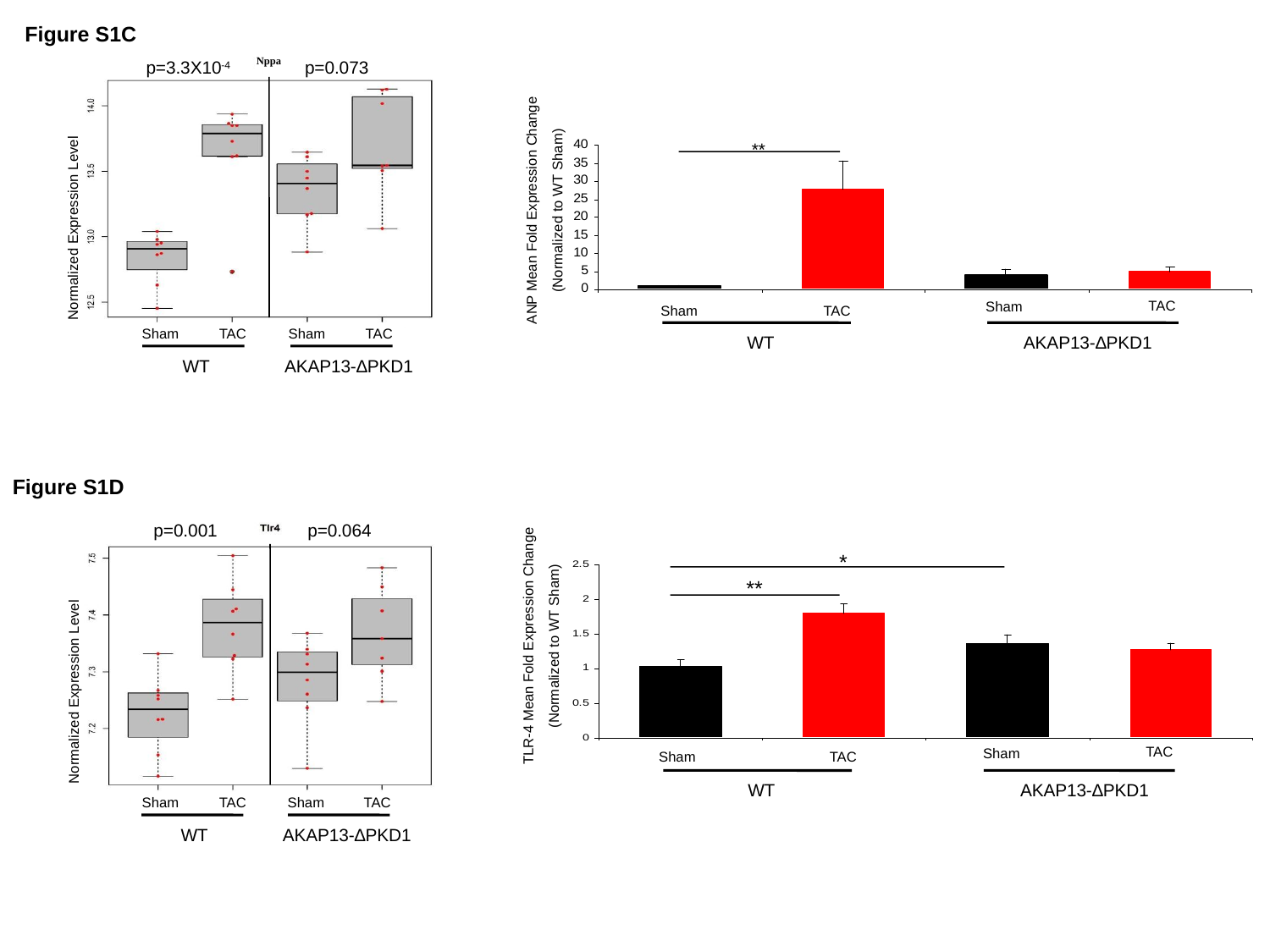

Figure S1C
Nppa
p=3.3X10-4
p=0.073
**
Normalized Expression Level
ANP Mean Fold Expression Change
(Normalized to WT Sham)
TAC
Sham
Sham
TAC
Sham
TAC
Sham
TAC
WT
AKAP13-∆PKD1
WT
AKAP13-∆PKD1
Figure S1D
p=0.001
p=0.064
*
**
TLR-4 Mean Fold Expression Change
(Normalized to WT Sham)
Normalized Expression Level
TAC
Sham
Sham
TAC
WT
AKAP13-∆PKD1
Sham
TAC
Sham
TAC
WT
AKAP13-∆PKD1
