# Supplementary material for: Genome-Wide Gene Expression Analysis Shows AKAP13-Mediated PKD1 Signaling Regulates the Transcriptional Response to Cardiac Hypertrophy
Source: PLoS One. 2015 Jul 20;10(7):e0132474. doi: 10.1371/journal.pone.0132474 (PMC4508115; doi:10.1371/journal.pone.0132474)
Supplement: S2 Fig — Protein levels of A) α-actinin and B) myosin heavy chain-β were significantly elevated in WT-TAC hearts, indicative of a normal hypertrophic response. These proteins were not significantly different in AKAP13-∆PKD1 sham/TAC hearts. (PPTX) [file pone.0132474.s002.pptx]

## Slide 1
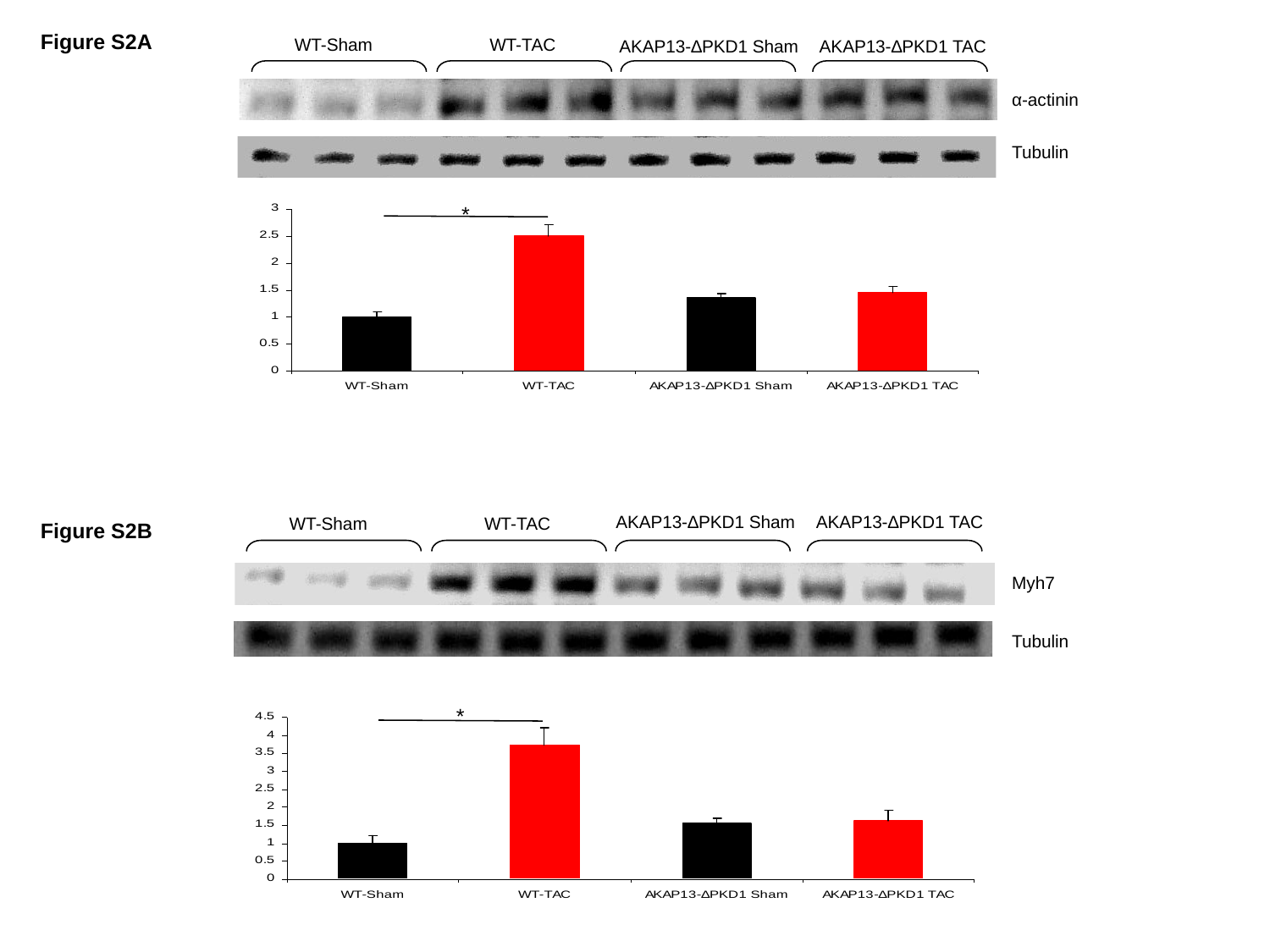

Figure S2A
WT-Sham
WT-TAC
AKAP13-∆PKD1 Sham
AKAP13-∆PKD1 TAC
α-actinin
Tubulin
*
AKAP13-∆PKD1 Sham
AKAP13-∆PKD1 TAC
WT-Sham
WT-TAC
Figure S2B
Myh7
Tubulin
*
