# Supplementary material for: Genome-Wide Gene Expression Analysis Shows AKAP13-Mediated PKD1 Signaling Regulates the Transcriptional Response to Cardiac Hypertrophy
Source: PLoS One. 2015 Jul 20;10(7):e0132474. doi: 10.1371/journal.pone.0132474 (PMC4508115; doi:10.1371/journal.pone.0132474)
Supplement: S3 Fig — Shown are expression changes determined via qPCR analysis for A) Aqp8, B) LMNA, C) MybpC2, D) NUAK1, E) Tgfβ-3, F) TnnT1, G) TnnT2, H) Ogfrl1. (PPTX) [file pone.0132474.s003.pptx]

## Slide 1
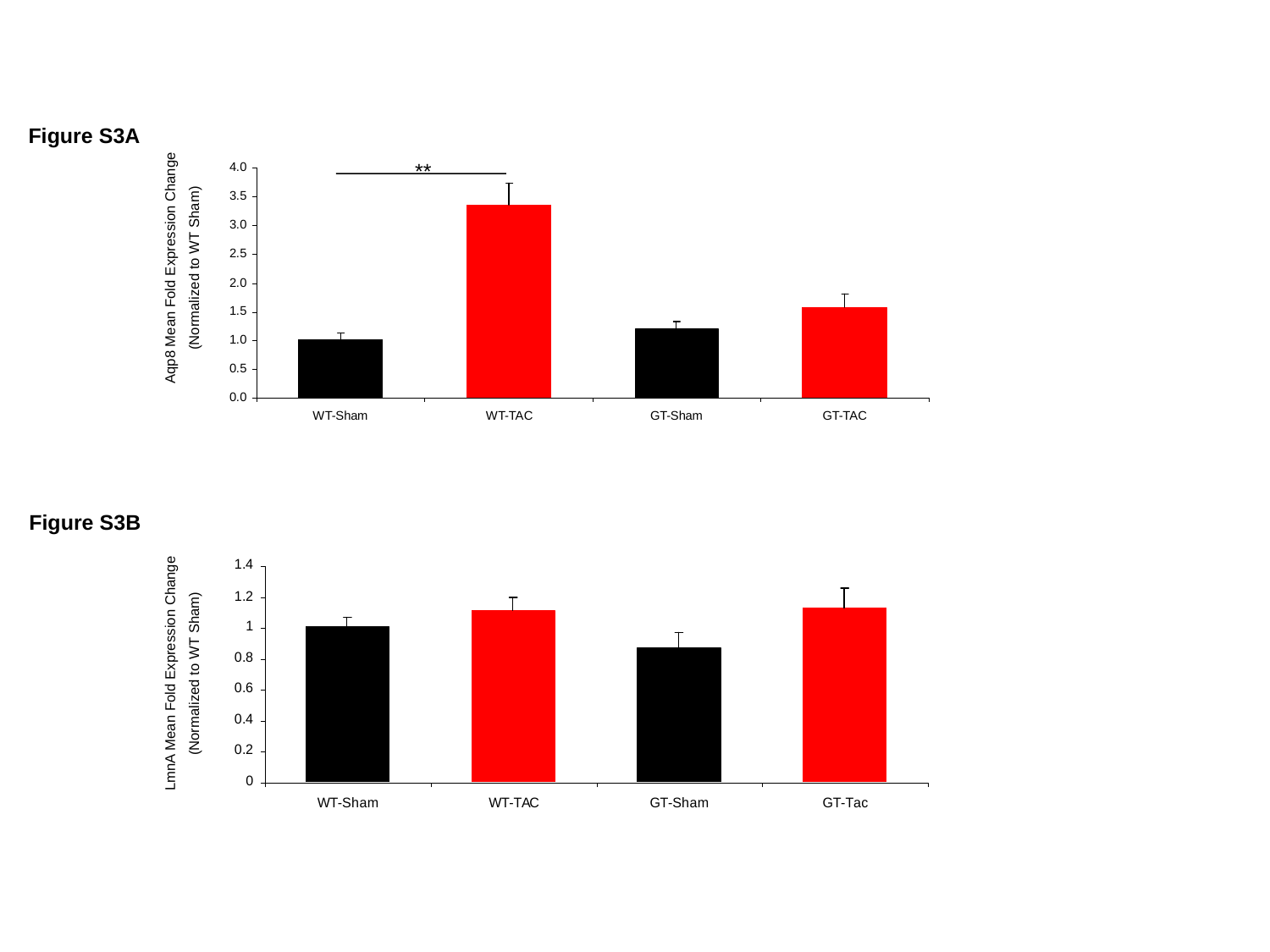

Figure S3A
**
Aqp8 Mean Fold Expression Change
(Normalized to WT Sham)
Figure S3B
LmnA Mean Fold Expression Change
(Normalized to WT Sham)

## Slide 2
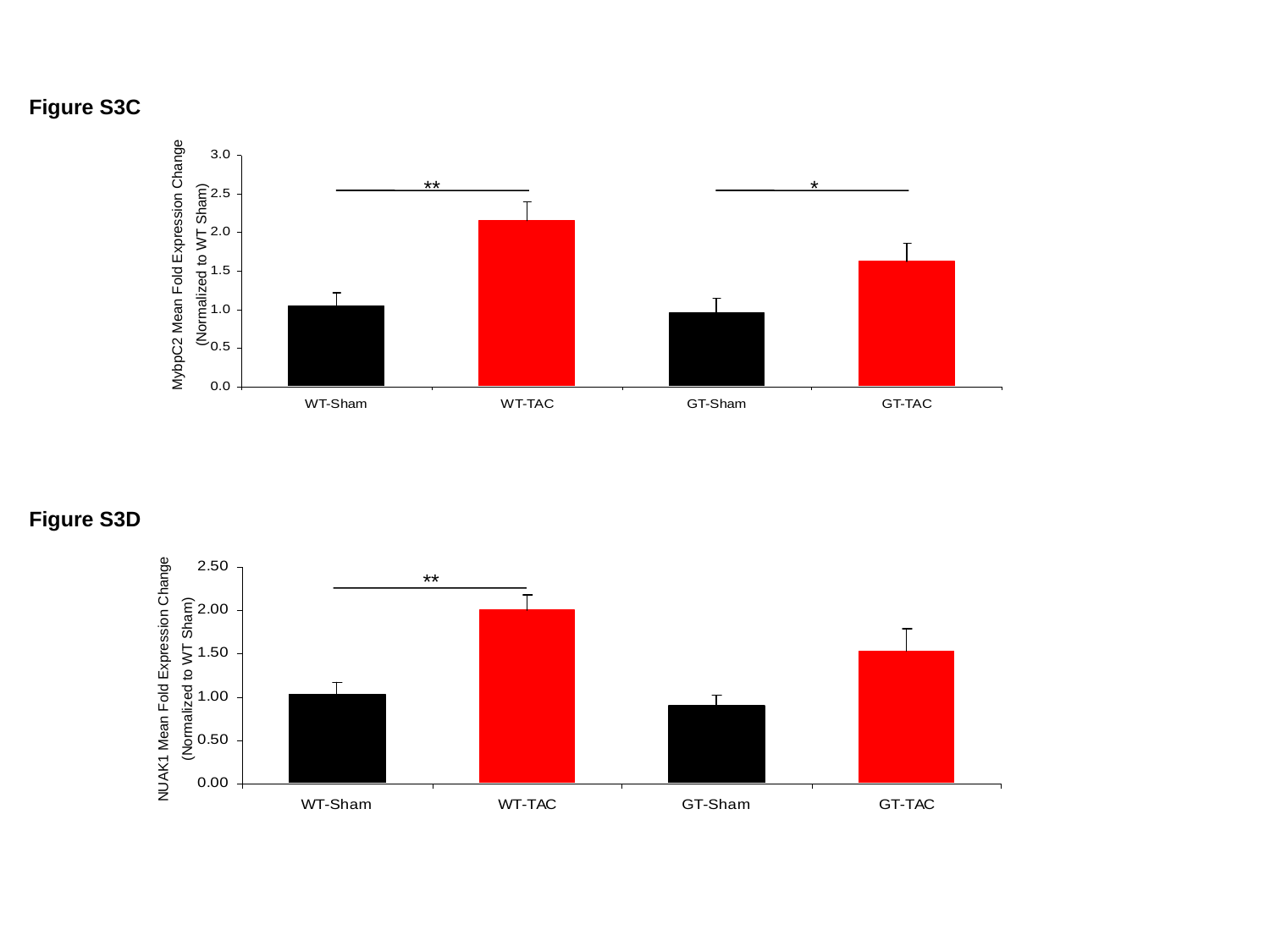

Figure S3C
**
*
MybpC2 Mean Fold Expression Change
(Normalized to WT Sham)
Figure S3D
**
NUAK1 Mean Fold Expression Change
(Normalized to WT Sham)

## Slide 3
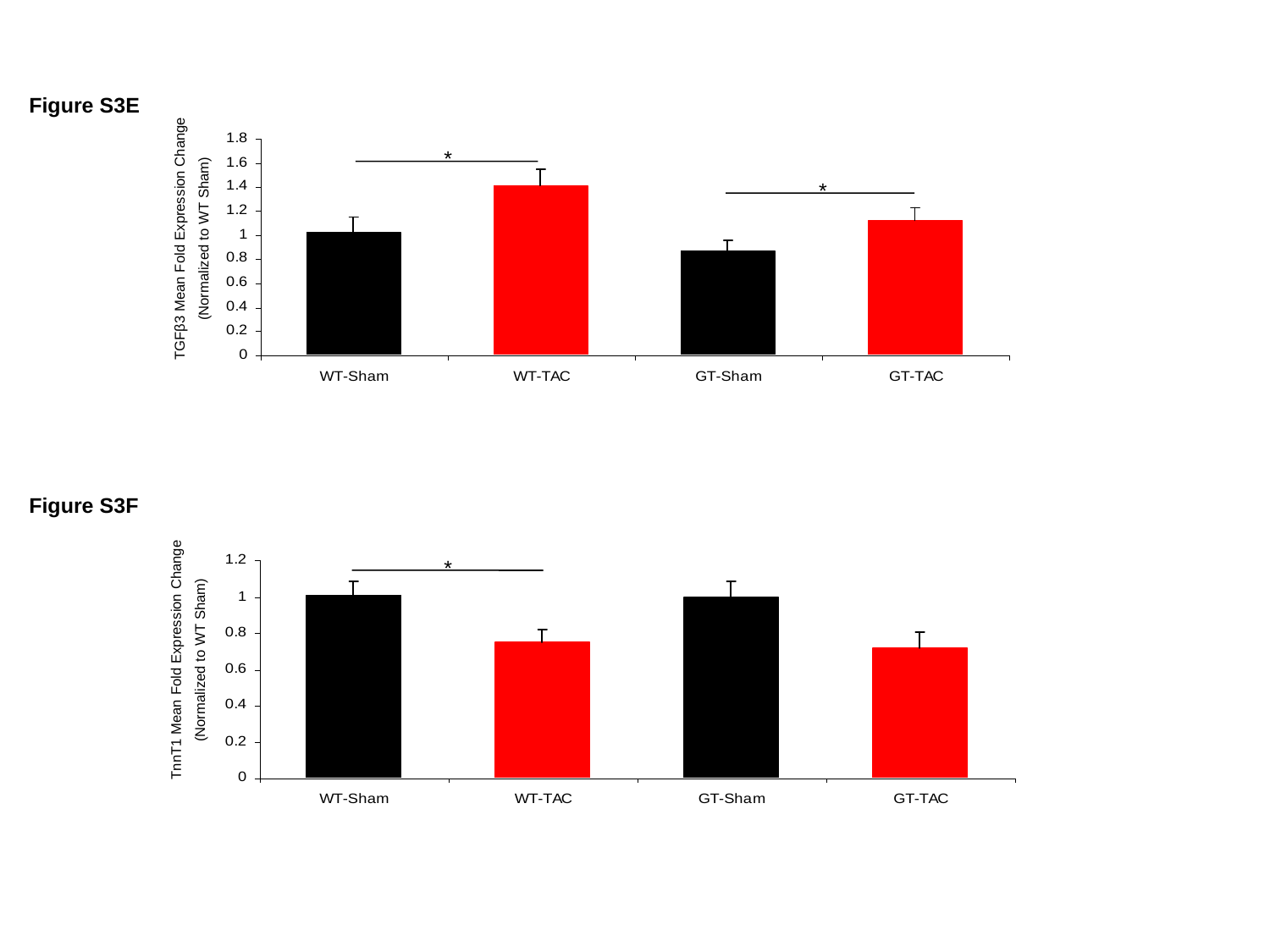

Figure S3E
*
*
TGFβ3 Mean Fold Expression Change
(Normalized to WT Sham)
Figure S3F
*
TnnT1 Mean Fold Expression Change
(Normalized to WT Sham)

## Slide 4
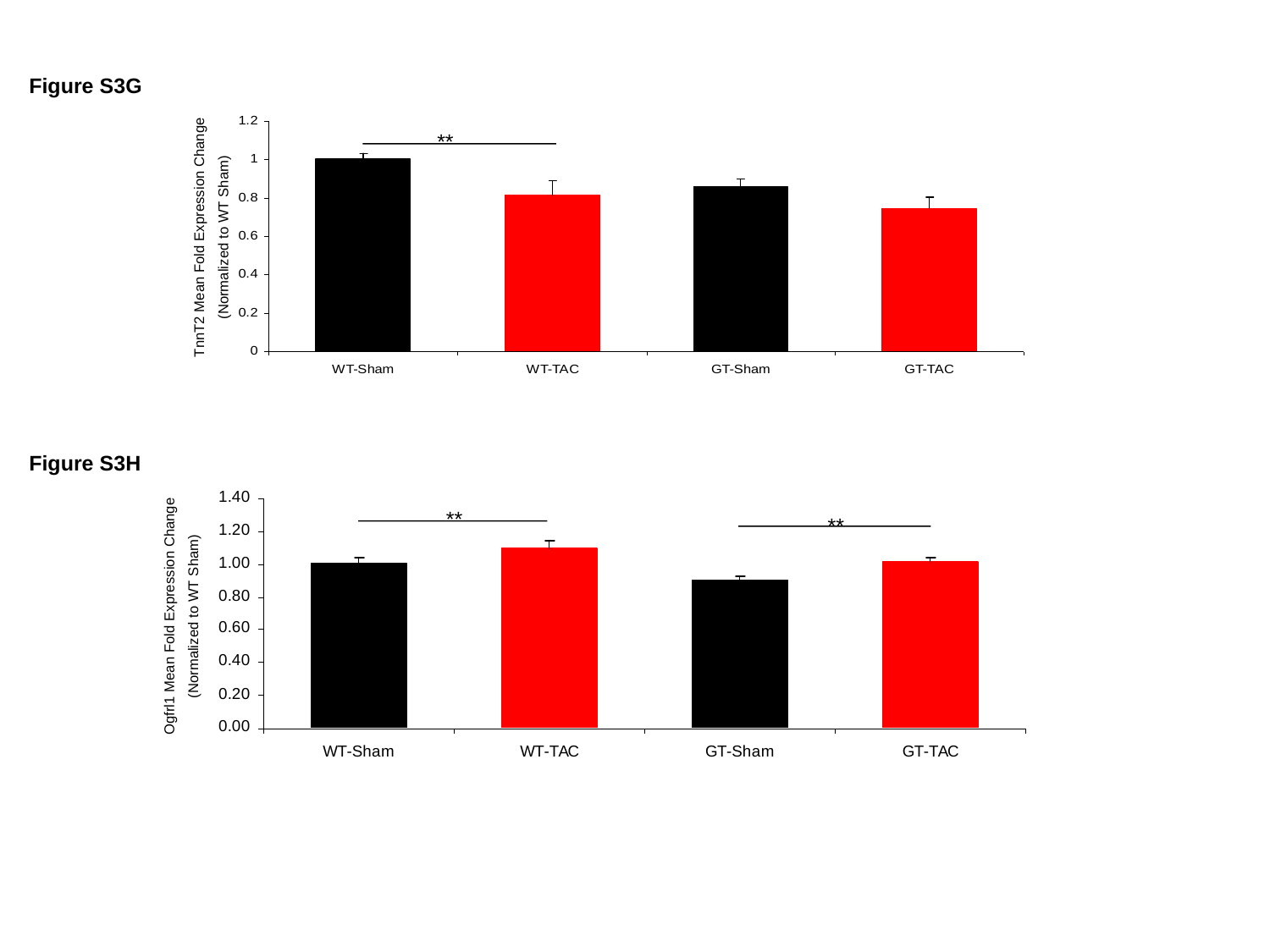

Figure S3G
**
TnnT2 Mean Fold Expression Change
(Normalized to WT Sham)
Figure S3H
**
**
Ogfrl1 Mean Fold Expression Change
(Normalized to WT Sham)
