# Supplementary material for: Genome-Wide Gene Expression Analysis Shows AKAP13-Mediated PKD1 Signaling Regulates the Transcriptional Response to Cardiac Hypertrophy
Source: PLoS One. 2015 Jul 20;10(7):e0132474. doi: 10.1371/journal.pone.0132474 (PMC4508115; doi:10.1371/journal.pone.0132474)
Supplement: S1 Table — (DOC) [file pone.0132474.s004.doc]

**Table S1.** Primers used in RT-PCR validation analyses.

| **Primer Name** | **Sequence** |
| --- | --- |
| Desmin For | GAGAGCAGGATCAACCTTCCTATCCAGAC |
| Desmin Rev | CTTTTTGGTATGGACTTCAGAACCCCTTTGCTC |
| MybpC2 For | CAGGCAAATGGTACAAGAACGGCGTGGA |
| MybpC2 Rev | GGCACGAATGTGTAGTCTCCCTCATCCT |
| TnnT1 For | TGGATTTTGATGACATCCACCGGAAGCGCAT |
| Tnnt1 Rev | TCGCCTCTCAATGCGGTCTTTTAGTGCAATGA |
| Mmus Fadd For | CAGGTGGCATTTGACATTGTGTGTGACAATGTG |
| Fadd Rev | AGCATTCTTCCAGACTTTCAGACTCTCCCTTAC |
| Nuak1 For | ATGCTGATGGTGAATCCCGATCGCAGAG |
| Nuak1 Rev | TGATGCCAATCGATGATCCGTGCCAAGAGT |
| TGFβ-3 For | GGATCTAGGCTGGAAATGGGTCCACGAA |
| TGFβ-3 Rev | TAGTCCAAGCACCGTGCTATGGGTTGTGT |
| TGFβR-3 For | CCGCAGAGAGAGGTTACCCTGCATCT |
| TGFβR-3 Rev | AACCCTCCGAAACCAGGAAGAGTCTGG |
| Aqp8 For | GATGTCTATCGGTCATTGAGAATAGTCCGAATACT |
| Aqp8 Rev | TTGAAGTGTCCACCGCTGATGTTCCCCAA |
| Lmna For | AAGTCGATGAAGAGGGAAAGTTCGTGCG |
| Lmna Rev | TGCCCAGCCTTTAGGGTGAACTTCGGT |
| Ogfrl1 For | AAGATGGAGAAGGGGAAGACCAGAGCAAAG |
| Ogfrl1 Rev | ACCAGAACTGGTTTGTGGGTTTGTGGCATTTTG |
| Tnnt2 For | CCAAGCCCAGCAGGCTCTTCATG |
| Tnnt2 Rev | CTTCCTGTTCTCGAAGTGAGCCTCGATCA |
| Tlr4 For | GAGGACTGGGTGAGAAATGAGCTGGTAAAGAATTTA |
| Tlr4 Rev | GGAATAAAGTCTCTGTAGTGAAGGCAGAGGTG |
