# Supplementary material for: Genome-Wide Gene Expression Analysis Shows AKAP13-Mediated PKD1 Signaling Regulates the Transcriptional Response to Cardiac Hypertrophy
Source: PLoS One. 2015 Jul 20;10(7):e0132474. doi: 10.1371/journal.pone.0132474 (PMC4508115; doi:10.1371/journal.pone.0132474)
Supplement: S3 Table — (DOC) [file pone.0132474.s006.doc]

| Name | p-value | Ratio |
| --- | --- | --- |
| Remodeling of Epithelial Adherens Junctions | 4.22X10^-8^ | 11/68 (.162) |
| NRF2-mediated Oxidative Stress Response | 2.33 X10^-7^ | 16/190 (0.084) |
| Epithelial Adherens Junction Signaling | 5.31X10^-7^ | 14/146 (0.096) |
| Glutathione-mediated Detoxification | 5.74 X10^-7^ | 7/28 (0.25) |
| Aryl Hydrocarbon Receptor Signaling | 1.11X10^-5^ | 12/148 (0.081) |

**SI Table 3.** Top Canonical Pathways.
