# Supplementary material for: Genome-Wide Gene Expression Analysis Shows AKAP13-Mediated PKD1 Signaling Regulates the Transcriptional Response to Cardiac Hypertrophy
Source: PLoS One. 2015 Jul 20;10(7):e0132474. doi: 10.1371/journal.pone.0132474 (PMC4508115; doi:10.1371/journal.pone.0132474)
Supplement: S4 Table — (DOC) [file pone.0132474.s007.doc]

**SI Table 4.** Top Upstream Regulators.

| Upstream Regulator | p-value of overlap |
| --- | --- |
| TGFβ-1 | 1.48X10^-11^ |
| TP53 | 1.09X10^-6^ |
| SOD1 | 3.05X10^-6^ |
| MAPT | 5.80X10^-6^ |
| AHR | 1.12X10^-5^ |
