# Supplementary material for: Genome-Wide Gene Expression Analysis Shows AKAP13-Mediated PKD1 Signaling Regulates the Transcriptional Response to Cardiac Hypertrophy
Source: PLoS One. 2015 Jul 20;10(7):e0132474. doi: 10.1371/journal.pone.0132474 (PMC4508115; doi:10.1371/journal.pone.0132474)
Supplement: S5 Table — (DOC) [file pone.0132474.s008.doc]

| Name | p-value | # molecules |
| --- | --- | --- |
| Cardiac Necrosis/Cell Death | 6.00X10^-4^ – 2.29X10^-1^ | 11 |
| Congenital Heart Anomaly | 6.76X10^-4^ – 3.11X10^-1^ | 5 |
| Cardiac Proliferation | 5.27X10^-3^ – 5.27X10^-3^ | 5 |
| Cardiac Stenosis | 6.69X10^-3^ – 7.17X10^-2^ | 2 |
| Cardiac Fibrosis | 1.43X10^-2^ – 2.44X10^-1^ | 8 |

**SI Table 5.** Top Toxicity Functions-Cardiotoxicity
